# Structural Diversity in Molecular Nickel Phosphide Carbonyl Nanoclusters

**DOI:** 10.1021/acs.inorgchem.0c02572

**Published:** 2020-10-21

**Authors:** Chiara Capacci, Cristiana Cesari, Cristina Femoni, Maria Carmela Iapalucci, Federica Mancini, Silvia Ruggieri, Stefano Zacchini

**Affiliations:** Dipartimento di Chimica Industriale “Toso Montanari”, Università di Bologna, Viale Risorgimento 4, 40136 Bologna, Italy

## Abstract

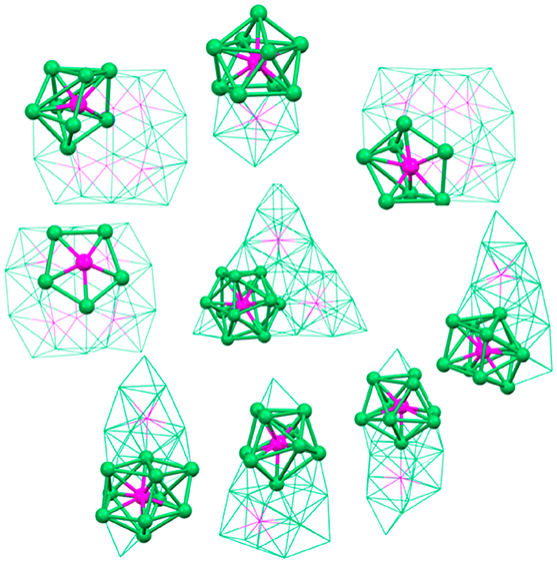

The reaction of [Ni_6_(CO)_12_]^2–^ as a [NBu_4_]^+^ salt in CH_2_Cl_2_ with 0.8 equiv of PCl_3_ afforded [Ni_14_P_2_(CO)_22_]^2–^. In contrast, the reactions of [Ni_6_(CO)_12_]^2–^ as a [NEt_4_]^+^ salt with 0.4–0.5 equiv of POCl_3_ afforded [Ni_22–*x*_P_2_(CO)_29–*x*_]^4–^ (*x* = 0.84) or [Ni_39_P_3_(CO)_44_]^6–^ by using CH_3_CN and thf as a solvent, respectively. Moreover, by using 0.7–0.9 mol of POCl_3_ per mole of [NEt_4_]_2_[Ni_6_(CO)_12_] both in CH_3_CN and thf, [Ni_23–*x*_P_2_(CO)_30–*x*_]^4–^ (*x* = 0.82) was obtained together with [Ni_22_P_6_(CO)_30_]^2–^ as a side product. [Ni_23–*x*_P_2_(CO)_30–*x*_]^4–^ (*x* = 0.82) and [Ni_22_P_6_(CO)_30_]^2–^ were separated owing to their different solubility in organic solvents. All the new molecular nickel phosphide carbonyl nanoclusters were structurally characterized through single crystal X-ray diffraction (SC-XRD) as [NBu_4_]_2_[Ni_14_P_2_(CO)_22_] (two different polymorphs, *P*2_1_/*n* and *C*2/*c*), [NEt_4_]_4_[Ni_23–*x*_P_2_(CO)_30–*x*_]·CH_3_COCH_3_·solv (*x* = 0.82), [NEt_4_]_2_[Ni_22_P_6_(CO)_30_]·2thf, [NEt_4_]_4_[Ni_22–*x*_P_2_(CO)_29–*x*_]·2CH_3_COCH_3_( *x* = 0.84) and [NEt_4_]_6_[Ni_39_P_3_(CO)_44_]·C_6_H_14_·solv. The metal cores’ sizes of these clusters range from 0.59 to 1.10 nm, and their overall dimensions including the CO ligands are 1.16–1.63 nm. In this respect, they are comparable to ultrasmall metal nanoparticles, molecular nanoclusters, or atomically precise metal nanoparticles. The environment of the P atoms within these molecular Ni–P–CO nanoclusters displays a rich diversity, that is, Ni_5_P pentagonal pyramid, Ni_7_P monocapped trigonal prism, Ni_8_P bicapped trigonal prism, Ni_9_P monocapped square antiprism, Ni_10_P sphenocorona, Ni_10_P bicapped square antiprism, and Ni_12_P icosahedron.

## Abstract 2

Five new molecular nickel phosphide carbonyl clusters have been obtained displaying overall sizes in the range 1.16−1.63 nm and very diverse environments for the phosphide atoms.

## Introduction

The Ni–P phase diagram is very rich, and 11 different phases have been identified, that is, Ni_3_P, Ni_8_P_3_, Ni_12_P_5_, Ni_2_P, Ni_5_P_4_, NiP (one monoclinic and two orthorhombic structures), NiP_2_ (cubic and monoclinic), and NiP_3_.^1−5^ Ni-rich phases (Ni_3_P, Ni_8_P_3_, Ni_12_P_5_, Ni_2_P) show isolated P atoms inside tricapped trigonal prismatic, monocapped square antiprismatic, cubic, or sphenocorona Ni cages. Conversely, direct P–P bonds are present in P-richer phases, affording P_2_, P_3_, and P_4_ units or longer chains.

Nickel phosphides, particularly as nanoparticles, are very interesting for applications in catalysis and electrocatalysis, in particular as alternatives to noble-metal catalysts.^6−13^ Ni_2_P supported on silica displays good activity in hydrodesulfurization (HDS) and hydrodenitrization (HDN).^14,15^ Ni_12_P_5_ nanoparticles have been demonstrated to be good catalysts in electrolytic and photoelectrolytic processes for hydrogen generation.^16,17^ Generally speaking, nickel phosphides are viewed as promising noble-metal-free catalysts for water splitting.^18−21^

Only two molecular Ni–P carbonyl nanoclusters have been characterized so far, that is, [Ni_11_P(CO)_18_]^3–^ and [H_6–*n*_Ni_31_P_4_(CO)_39_]^*n*−^ (*n* = 4, 5).^22^ The unique P atom of [Ni_11_P(CO)_18_]^3–^ is enclosed within a Ni_10_ spheonocorona cage, whereas [H_6–*n*_Ni_31_P_4_(CO)_39_]^*n*−^ (*n* = 4, 5) contains two distorted Ni_9_P monocapped square antiprisms and two distorted Ni_10_P bicapped square antiprisms. [H_6–*n*_Ni_31_P_4_(CO)_39_]^*n*−^ (*n* = 4, 5) also represents the largest structurally characterized metal carbonyl cluster containing P atoms. Indeed, several Co, Rh, Ru, and Os phosphide carbonyl clusters are known, but with a nuclearity of 6–10 and containing 1–2 P atoms.^23−30^ Due to the larger radius of Os compared to Rh and Ru, phosphorus is enclosed within a trigonal prismatic cage in the case of Os clusters, whereas it requires larger square antiprismatic cages for Rh and Ru. Because of the even smaller size of cobalt, P atoms may be both fully interstitial (within capped square antiprismatic cages) and semi-interstitial.

Aiming at widening the scope of our work, we have attempted synthesis and structural characterization by single-crystal X-ray diffraction (SC-XRD) of other nickel phosphide carbonyl nanoclusters. Herein, we present the new fully interstitial polyphosphides [Ni_14_P_2_(CO)_22_]^2–^, [Ni_23–*x*_P_2_(CO)_30–*x*_]^4–^ (*x* = 0.82), [Ni_22–*x*_P_2_(CO)_29–*x*_]^4–^ (*x* = 0.84), and [Ni_39_P_3_(CO)_44_]^6–^, as well as the [Ni_22_P_6_(CO)_30_]^2–^ cluster, which contains fully interstitial, semi-interstitial, and exposed P atoms. The structural diversity of these Ni–P nanoclusters is discussed.

## Experimental Section

### General Procedures

All reactions and sample manipulations were carried out using standard Schlenk techniques under nitrogen and in dried solvents. All of the reagents were commercial products (Aldrich) of the highest purity available and used as received, except [NR_4_]_2_[Ni_6_(CO)_12_] (R = Et, Bu), which has been prepared according to the literature.^31^ Analysis of Ni was performed by atomic absorption on a Pye-Unicam instrument. Analyses of C, H, and N were obtained with a Thermo Quest Flash EA 1112NC instrument. IR spectra were recorded on a PerkinElmer Spectrum One interferometer in CaF_2_ cells. ^31^P{^1^H} NMR measurements were performed on a Varian Mercury Plus 400 MHz instrument. The phosphorus chemical shifts were referenced to external H_3_PO_4_ (85% in D_2_O). Structure drawings have been performed with SCHAKAL99^32^ and Mercury 2020.1.^33^

***Warning!** CO and Ni(CO)_4_ may be generated during manipulation of these compounds. All of the operations must be carried out under a well-ventilated fume hood.*

### Synthesis of [NBu_4_]_2_[Ni_14_P_2_(CO)_22_]

A solution of PCl_3_ (0.182 g, 1.33 mmol) in CH_2_Cl_2_ (30 mL) was added to a solution of [NBu_4_]_2_[Ni_6_(CO)_12_] (1.65 g, 1.49 mmol) in CH_2_Cl_2_ (20 mL) over a period of 4 h. The resulting mixture was stirred at room temperature for 1 h and, then, the solvent removed *in vacuo*. The residue was washed with H_2_O (3 × 20 mL) and extracted with CH_2_Cl_2_ (20 mL). Crystals of [NBu_4_]_2_[Ni_14_P_2_(CO)_22_] suitable for X-ray analyses were obtained by layering *n*-hexane (40 mL) on the CH_2_Cl solution (yield 0.70 g, 55% based on Ni). Two different polymorphs, space groups *P*2_1_/*n* and *C*2/*c*, were obtained.

C_54_H_72_N_2_Ni_14_O_22_P_2_(1985.02), calcd.: C 32.84, H 3.68, N 1.42. Found: C 32.57, H 3.89, N 1.19. IR (CH_2_Cl_2_, 293 K), ν_CO_: 2032(s), 1862(m) cm^–1^. IR (CH_3_CN, 293 K), ν_CO_: 2026(s), 1863(m) cm^–1^.

### Synthesis of [NEt_4_]_4_[Ni_23–*x*_P_2_(CO)_30–*x*_]·CH_3_COCH_3_·solv (*x* = 0.82)

A solution of POCl_3_ (0.310 g, 2.02 mmol) in CH_3_CN (15 mL) was added to a solution of [NEt_4_]_2_[Ni_6_(CO)_12_] (2.39 g, 2.52 mmol) in CH_3_CN (50 mL) over a period of 4 h. The resulting mixture was stirred at room temperature for 1 h and, then, the solvent removed *in vacuo*. The residue was washed with H_2_O (3 × 20 mL), thf (3 × 20 mL), and extracted with CH_3_COCH_3_ (20 mL). Crystals of [NEt_4_]_4_[Ni_23–*x*_P_2_(CO)_30–*x*_]·CH_3_COCH_3_·solv (*x* = 0.82) suitable for X-ray analyses were obtained by layering *n*-hexane (40 mL) on the acetone solution (yield 0.73 g, 39% based on Ni).

C_64.18_H_86_N_4_Ni_22.18_O_30.18_P_2_ (2760.97), calcd.: C 28.08, H 3.16, N 2.04. Found: C 27.85, H 3.33, N 1.84. IR (CH_3_CN, 293 K) ν_CO_: 2004(s), 1865(ms) cm^–1^.

### Synthesis of [NEt_4_]_2_[Ni_22_P_6_(CO)_30_]·2thf

A solution of POCl_3_ (0.284 g, 1.85 mmol) in thf (15 mL) was added to a solution of [NEt_4_]_2_[Ni_6_(CO)_12_] (2.42 g, 2.55 mmol) in thf (50 mL) over a period of 4 h. The resulting mixture was stirred at room temperature for 1 h and, then, the solvent removed *in vacuo*. The residue was washed with H_2_O (3 × 20 mL) and extracted with thf (20 mL). [The residue not soluble in thf was further extracted with acetone (20 mL). The IR spectrum of the acetone solution is identical to that of [NEt_4_]_4_[Ni_23–*x*_P_2_(CO)_30–*x*_]·2CH_3_COCH_3_.] Crystals of [NEt_4_]_2_[Ni_22_P_6_(CO)_30_]·2thf suitable for X-ray analyses were obtained by layering *n*-hexane (40 mL) on the thf solution (yield 0.15 g, 8% based on Ni).

C_54_H_56_N_2_Ni_22_O_32_P_6_ (2722.44), calcd.: C 23.96, H 2.09, N 1.04. Found: C 23.79, H 2.21, N 0.88. IR (nujol, 293 K), ν_CO_: 2032(vs), 1991(ms), 1931(m), 1844(m), 1825(m) cm^–1^. IR (thf, 293 K), ν_CO_: 2031(vs), 1943(w), 1836(m) cm^–1^.

### Synthesis of [NEt_4_]_4_[Ni_22–*x*_P_2_(CO)_29–*x*_]·2CH_3_COCH_3_ (*x* = 0.84)

A solution of POCl_3_ (0.144 g, 0.941 mmol) in CH_3_CN (5 mL) was added to a solution of [NEt_4_]_2_[Ni_6_(CO)_12_] (2.16 g, 2.28 mmol) in CH_3_CN (50 mL) over a period of 4 h. The resulting mixture was stirred at room temperature for 1 h and, then, the solvent removed *in vacuo*. The residue was washed with H_2_O (3 × 20 mL), thf (3 × 20 mL), and extracted with CH_3_COCH_3_ (20 mL). Crystals of [NEt_4_]_4_[Ni_22–*x*_P_2_(CO)_29–*x*_]·2CH_3_COCH_3_ (*x* = 0.84) suitable for X-ray analyses were obtained by layering *n*-hexane (40 mL) on the acetone solution (yield 0.63 g, 36% based on Ni).

C_66.16_H_92_N_4_Ni_21.16_O_30.16_P_2_ (2730.37), calcd.: C 29.26, H 3.42, N 2.06. Found: C 29.04, H 3.61, N 1.79. IR (nujol, 293 K), ν_CO_: 2026(sh), 1993(vs), 1966(m), 1948(sh), 1834(s) cm^–1^. IR (acetone, 293 K), ν_CO_: 1997(vs), 1963(sh), 1870(s) cm^–1^. IR (CH_3_CN, 293 K), ν_CO_: 2003(vs), 1962(sh), 1869(s) cm^–1^. IR (dmso, 293 K), ν_CO_: 1993(vs), 1960(sh), 1863(s) cm^–1^.

### Synthesis of [NEt_4_]_6_[Ni_39_P_3_(CO)_44_]·C_6_H_14_·solv

A solution of POCl_3_ (0.193 g, 1.26 mmol) in thf (20 mL) was added to a solution of [NEt_4_]_2_[Ni_6_(CO)_12_] (2.39 g, 2.52 mmol) in thf (50 mL) over a period of 4 h. The resulting mixture was stirred at room temperature for 1 h and, then, the solvent removed *in vacuo*. The residue was washed with H_2_O (3 × 20 mL), thf (3 × 20 mL), and CH_3_COCH_3_ (3 × 20 mL) and extracted with CH_3_CN (20 mL). Crystals of [NEt_4_]_6_[Ni_39_P_3_(CO)_44_]·C_6_H_14_·solv suitable for X-ray analyses were obtained by layering *n*-hexane (2 mL) and di-iso-propyl-ether (40 mL) on the CH_3_CN solution (yield 0.18 g, 10% based on Ni).

C_98_H_134_N_6_Ni_39_O_44_P_3_ (4482.70), calcd.: C 26.42, H 3.03, N 1.89. Found: C 26.61, H 3.22, N 1.68, Ni 46.04. IR (CH_3_CN, 293 K), ν_CO_: 1998(vs), 1868(s) cm^–1^.

### X-ray Crystallographic Study

Crystal data and collection details for [NBu_4_]_2_[Ni_14_P_2_(CO)_22_] (*P*2_1_/*n*), [NBu_4_]_2_[Ni_14_P_2_(CO)_22_] (*C*2/*c*), [NEt_4_]_4_[Ni_23–*x*_P_2_(CO)_30–*x*_]·CH_3_COCH_3_·solv (*x* = 0.82), [NEt_4_]_2_[Ni_22_P_6_(CO)_30_]·2thf, [NEt_4_]_4_[Ni_22–*x*_P_2_(CO)_29–*x*_]·2CH_3_COCH_3_ (*x* = 0.84), and [NEt_4_]_6_[Ni_39_P_3_(CO)_44_]·C_6_H_14_·solv are reported in Table S1 in the Supporting Information. ORTEP drawings of all the structures are included in Figures S5–S9 in the Supporting Information. The diffraction experiments were carried out on a Bruker APEX II diffractometer equipped with a CCD detector ([NBu_4_]_2_[Ni_14_P_2_(CO)_22_] (*P*2_1_/*n*), [NBu_4_]_2_[Ni_14_P_2_(CO)_22_] (*C*2/*c*), [NEt_4_]_4_[Ni_23–*x*_P_2_(CO)_30–*x*_]·CH_3_COCH_3_·solv (*x* = 0.82), [NEt_4_]_6_[Ni_39_P_3_(CO)_44_]·C_6_H_14_·solv), or a PHOTON2 detector ([NEt_4_]_4_[Ni_22–*x*_P_2_(CO)_29–*x*_]·2CH_3_COCH_3_ (*x* = 0.84) and [NEt_4_]_2_[Ni_22_P_6_(CO)_30_]·2thf) using Mo Kα radiation. Data were corrected for Lorentz polarization and absorption effects (empirical absorption correction SADABS).^34^ Structures were solved by direct methods and refined by full-matrix least-squares based on all data using *F*^2^.^35^ Hydrogen atoms were fixed at calculated positions and refined by a riding model. All non-hydrogen atoms were refined with anisotropic displacement parameters, unless otherwise stated. Further details are given in the Supporting Information.

## Results and Discussion

**Scheme 1:**
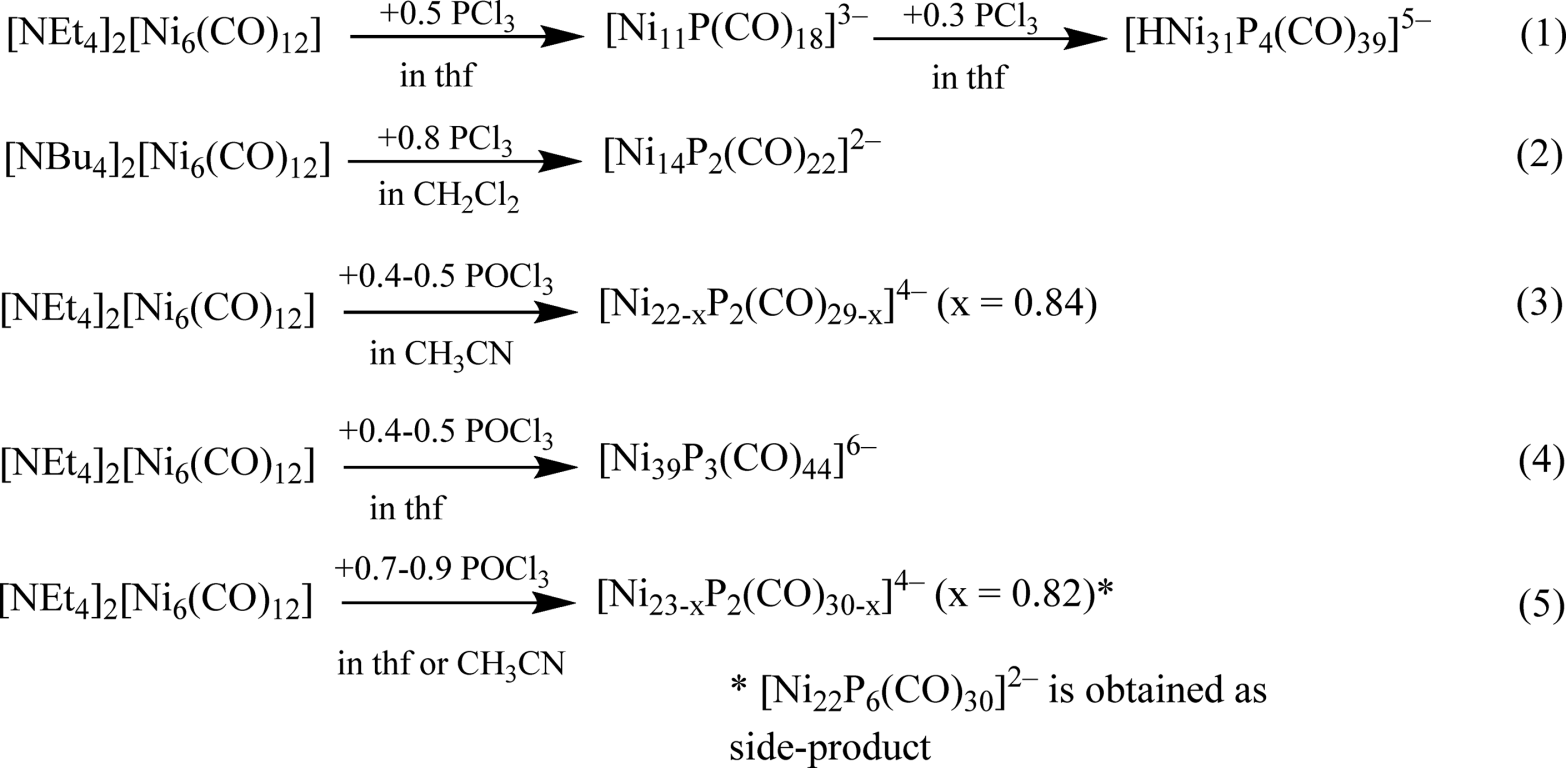
Synthesis of Ni–P–CO Clusters

The synthesis of Ni–P–CO clusters is very sensitive to experimental conditions (Scheme 1), that is, the stoichiometric ratio, counterion, solvent, and P source. Thus, by reacting [Ni_6_(CO)_12_]^2–^ as a [NEt_4_]^+^ salt with PCl_3_ in thf, [Ni_11_P(CO)_18_]^3–^ and, then, [HNi_31_P_4_(CO)_39_]^5–^ were formed in sequence, as previously reported.^22^ Conversely, carrying out a similar reaction in CH_2_Cl_2_ with [NBu_4_]^+^ as a counterion, [Ni_14_P_2_(CO)_22_]^2–^ was obtained. When POCl_3_ was used instead of PCl_3_, the products observed were [Ni_22–*x*_P_2_(CO)_29–*x*_]^4–^ (*x* = 0.84) by using 0.4–0.5 mol of POCl_3_ per mole of [NEt_4_]_2_[Ni_6_(CO)_12_]^2–^ in CH_3_CN or [Ni_39_P_3_(CO)_44_]^6–^ by performing the same reaction in thf. In contrast, [Ni_23–*x*_P_2_(CO)_30–*x*_]^4–^ (*x* = 0.82) was obtained by using 0.7–0.9 mol of POCl_3_ per mole of [NEt_4_]_2_[Ni_6_(CO)_12_]^2–^ in CH_3_CN or thf. In the latter case, [Ni_22_P_6_(CO)_30_]^2–^ was also observed as a side product. Details on the syntheses and characterizations of the new clusters [Ni_14_P_2_(CO)_22_]^2–^, [Ni_22–*x*_P_2_(CO)_29–*x*_]^4–^ (*x* = 0.84), [Ni_39_P_3_(CO)_44_]^6–^, [Ni_23–*x*_P_2_(CO)_30–*x*_]^4–^ (*x* = 0.82), and [Ni_22_P_6_(CO)_30_]^2–^ are reported in the following sections.

### Synthesis and Molecular Structure of [Ni_14_P_2_(CO)_22_]^2–^

The reaction of [Ni_6_(CO)_12_]^2–^ as a [NBu_4_]^+^ salt in CH_2_Cl_2_ with PCl_3_ afforded [NBu_4_]_2_[Ni_14_P_2_(CO)_22_] as an oily precipitate. Ni(CO)_4_ was formed as a side product, as inferred by IR spectroscopy, and eliminated in a vacuum. The solid residue was recovered after filtration and washed with H_2_O and [Ni_14_P_2_(CO)_22_]^2–^ extracted in CH_2_Cl_2_. Crystals of [NBu_4_]_2_[Ni_14_P_2_(CO)_22_] suitable for SC-XRD were grown by slow diffusion of *n*-hexane pn the CH_2_Cl_2_ solution. Two different polymorphs of [NBu_4_]_2_[Ni_14_P_2_(CO)_22_] were obtained (monoclinic *P*2_1_/*n* and monoclinic *C*2/*c*).

Crystals of [NBu_4_]_2_[Ni_14_P_2_(CO)_22_] displayed ν_CO_ at 2032(s) and 1862(m) cm^–1^ in CH_2_Cl_2_ solution and ν_CO_ at 2025(s) and 1863(m) cm^–1^ in CH_3_CN solution.

**Figure 1:**
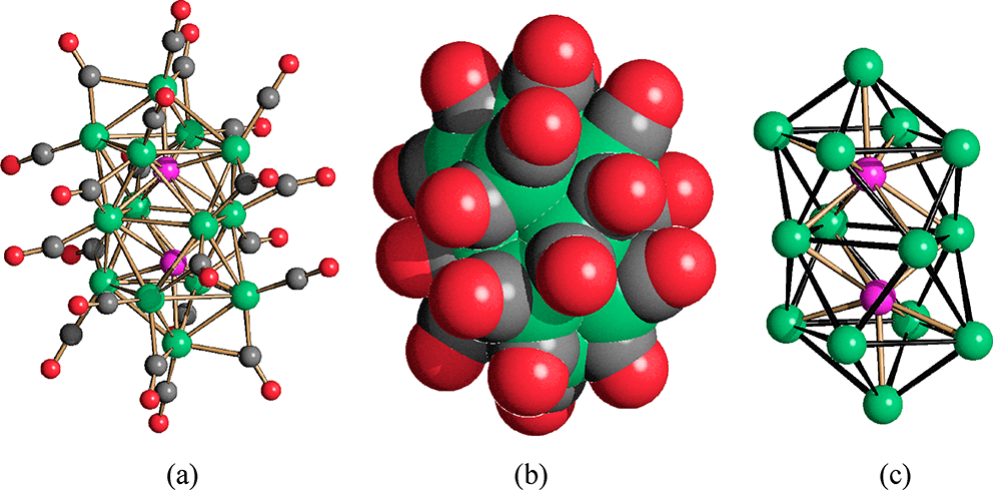
(a) The molecular structure of [Ni_14_P_2_(CO)_22_]^2–^; (b) its space-filling model; (c) the Ni_14_P_2_ core (Ni, green; P, purple; C, gray; O, red). The Ni–Ni bonds of the Ni_14_ cage are represented in black in c.

The metal cage of [Ni_14_P_2_(CO)_22_]^2–^ consists of two monocapped P-centered square-antiprismatic Ni_9_P units fused through a common square face (Figure 1, Table 1). A similar environment was displayed by two P atoms within the larger [H_6–*n*_Ni_31_P_4_(CO)_39_]^*n*−^ (*n* = 4, 5) cluster,^22^ whereas the other two P atoms were encapsulated within Ni_10_P bicapped square antiprisms. Conversely, [Ni_11_P(CO)_18_]^3–^ presents a Ni_10_P sphenocorona cage. It should be noticed that square-antiprismatic cages (with the possibility of capping atoms) were previously found in the case of fully interstitial carbonyl monophosphide clusters of Ru, Rh, and Co.^23−28^ A monocapped square-antiprismatic environment was found also in the Ni_3_P phase.^1−3^

**Table 1:** Main Bond Distances (Å) of [Ni_14_P_2_(CO)_22_]^2–^, [Ni_23–*x*_P_2_(CO)_30–*x*_]^4–^, [Ni_22–*x*_P_2_(CO)_29–*x*_]^4–^, [Ni_22_P_6_(CO)_30_]^2–^, and [Ni_39_P_3_(CO)_44_]^3–^

	Ni–Ni	Ni–P	P···P
[Ni_14_P_2_(CO)_22_]^2–,^^a^	2.4242(9)–3.0530(10)	2.1976(14)–2.5308(13)	2.641(2)
	average 2.689(4)	average 2.294(4)	
[Ni_14_P_2_(CO)_22_]^2–,^^b^	2.4248(17)–3.051(2)	2.204(3)–2.547(3)	2.664(5)
	average 2.691(8)	average 2.295(9)	
[Ni_23–*x*_P_2_(CO)_30–*x*_]^4–^	2.3254(11)–2.8235(11)	2.1681(19)–2.5117(18)	
	average 2.591(9)	average 2.332(8)	
[Ni_22–*x*_P_2_(CO)_29–*x*_]^4–^	2.280(14)–2.963(3)	2.119(4)–2.446(4)	
	average 2.60(2)	average 2.296(16)	
[Ni_22_P_6_(CO)_30_]^2–^	2.448(3)–2.869(4)	2.200(4)–2.394(4)	
	average 2.61(2)	average 2.29(2)	
[Ni_39_P_3_(CO)_44_]^3–^	2.312(3)–2.8733(16)	2.291(3)–2.678(2)	
	average 2.584(12)	average 2.452(7)	

a As found in [NBu_4_]_2_[Ni_14_P_2_(CO)_22_], *P*2_1_/*n*.

b As found in [NBu_4_]_2_[Ni_14_P_2_(CO)_22_], *C*2/*c*.

The Ni–Ni [2.4242(9)–3.0530(10) Å, average 2.689(4) Å for polymorph *P*2_1_/*n*; 2.4248(17)–3.051(2) Å, average 2.691(8) Å for polymorph *C*2/*c*] and Ni–P [2.1976(14)–2.5308(13) Å, average 2.294(4) Å for polymorph *P*2_1_/*n*; 2.204(3)–2.547(3) Å, average 2.295(9) Å for polymorph *C*2/*c*] bonding distances are similar to other Ni–P carbonyl clusters.^22^ The P···P contact [2.641(2) Å for polymorph *P*2_1_/*n*; 2.664(5) Å for polymorph *C*2/*c*] is essentially nonbonding. Indeed, the covalent and van der Waals radii of phosphorus are 1.11 and 1.80 Å, respectively.^36^ The cluster contains 22 carbonyl ligands, 10 in terminal and 14 in edge bridging positions.

The cluster possesses 196 cluster valence electrons [CVE; 14 × 10 (Ni) + 5 × 2 (P) + 22 × 2 (CO) + 2 (charge)] which correspond to 6*n* + 14 cluster molecular orbitals (CMOs). This electron count is in accord with the Mingos fused formalism, since the cluster results from two monocapped square-antiprisms (130 CVE based on Wade-Mingos rules) fused through a square face (64 CVE): 130 × 2 – 64 = 196 CVE.^37^ For comparison, both the monocapped square antiprismatic clusters [Ni_9_C(CO)_17_]^2–^^38^ and [Rh_9_P(CO)_21_]^2–^^27^ possess 130 CVE.

**Figure 2:**
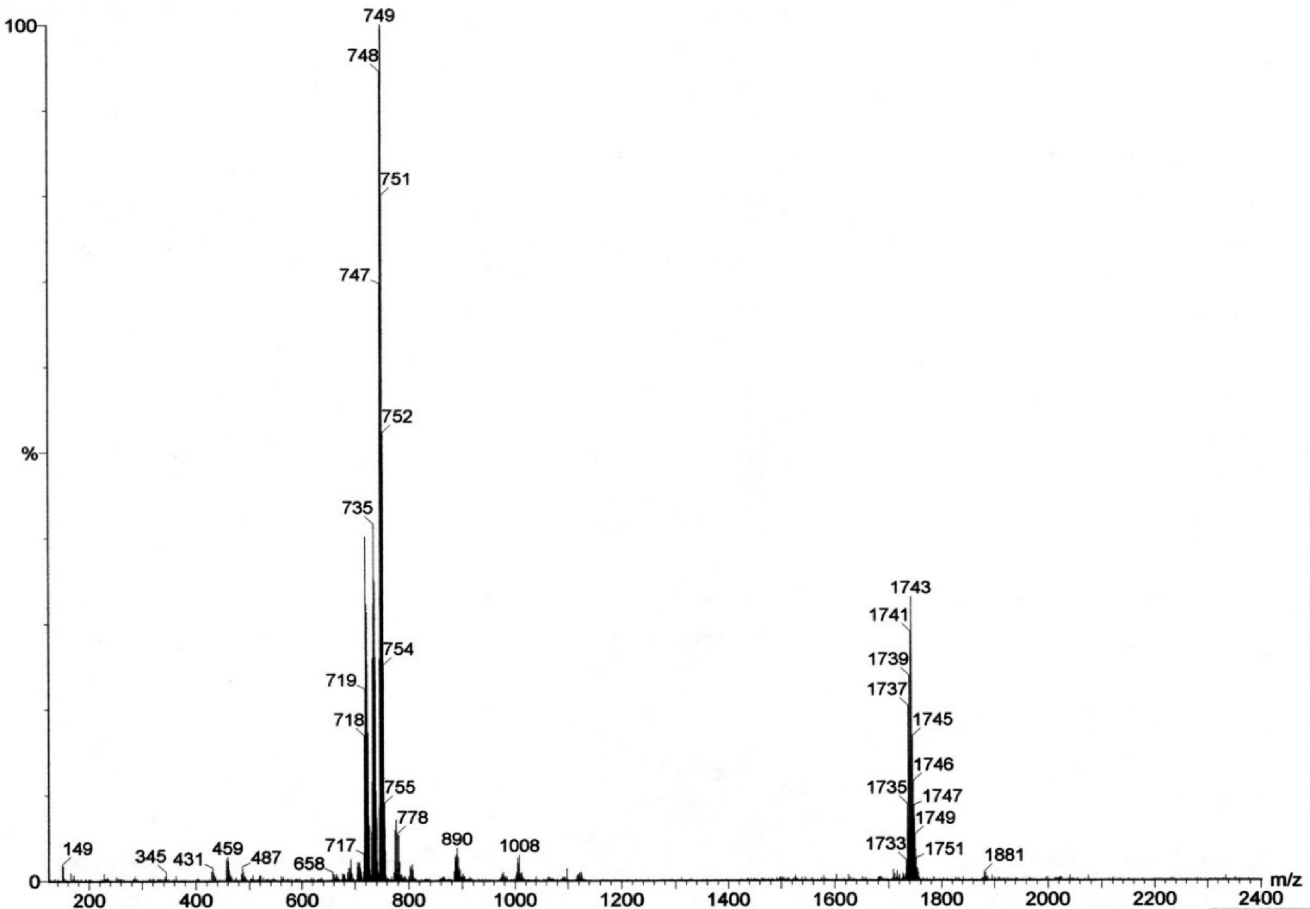
ESI-MS spectrum in CH_3_CN (ES−) of [NBu_4_]_2_[Ni_14_P_2_(CO)_22_].

The ESI-MS spectrum of [Ni_14_P_2_(CO)_22_]^2–^ in CH_3_CN solution is reported in Figure 2. The strongest peak at *m*/*z* 749 corresponds to the molecular ion [Ni_14_P_2_(CO)_22_]^2–^ confirming the fact that the cluster fully retains its nature in solution. The dianionic charge of the cluster is further corroborated by the stepwise loss of 14 uma corresponding to a CO ligand (peaks at *m*/*z* 735 and 721) for a dianionic species. Finally, the peak at *m*/*z* 1743 is due to the {[Ni_14_P_2_(CO)_22_][NBu_4_]}^−^ adduct.

[Ni_14_P_2_(CO)_22_]^2–^ is poorly stable in solution for a prolonged time. Indeed, while attempting to record its ^31^P{^1^H} NMR spectrum overnight, several resonances appeared in the range 100–550 ppm, suggesting extended decomposition (Figure S1 in Supporting Information). It must be remarked that phosphorus resonances of interstitial phosphide in metal carbonyl clusters are reported in a very large chemical-shift range, that is, 88–775 ppm.^27,30,39−41^ The IR spectrum recorded after the overnight ^31^P{^1^H} NMR spectrum is rather broad, in keeping with the presence of a mixture of decomposition products. The ν_CO_ bands (2005(s), 1863(ms) cm^–1^) are indicative of larger clusters, suggesting that the decomposition of [Ni_14_P_2_(CO)_22_]^2–^ involves some condensation processes. Unfortunately, it has not been possible to isolate and structurally characterize such products.

### Synthesis and Molecular Structures of [Ni_23–*x*_P_2_(CO)_30–*x*_]^4–^ (*x* = 0.82) and [Ni_22_P_6_(CO)_30_]^2–^

The new cluster [Ni_23–*x*_P_2_(CO)_30–*x*_]^4–^ (*x* = 0.82) was obtained from the reaction of [NEt_4_]_2_[Ni_6_(CO)_12_] with 0.7–0.9 equiv of POCl_3_ in CH_3_CN or thf. The formation of [Ni_23–*x*_P_2_(CO)_30–*x*_]^4–^ (*x* = 0.82) was accompanied by traces of the new [Ni_22_P_6_(CO)_30_]^2–^ cluster. The two species were separated since [Ni_22_P_6_(CO)_30_]^2–^ was soluble in thf, whereas [Ni_23–*x*_P_2_(CO)_30–*x*_]^4–^ (*x* = 0.82) was soluble in acetone.

**Figure 3:**
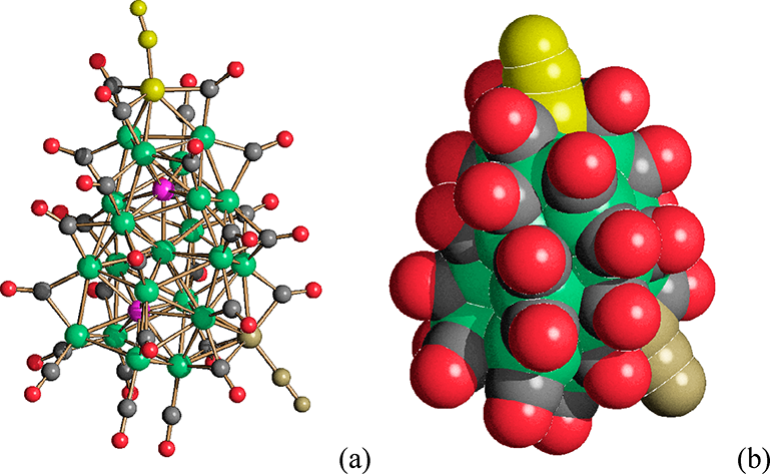
(a) The molecular structure of [Ni_23–*x*_P_2_(CO)_30–*x*_]^4–^ (*x* = 0.82) and (b) its space-filling model (Ni, green; P, purple; C, gray; O, red). The Ni(CO) fragment with 0.50 occupancy factor is represented in yellow. The Ni(CO) fragment with 0.68 occupancy factor is represented in olive green.

**Figure 4:**
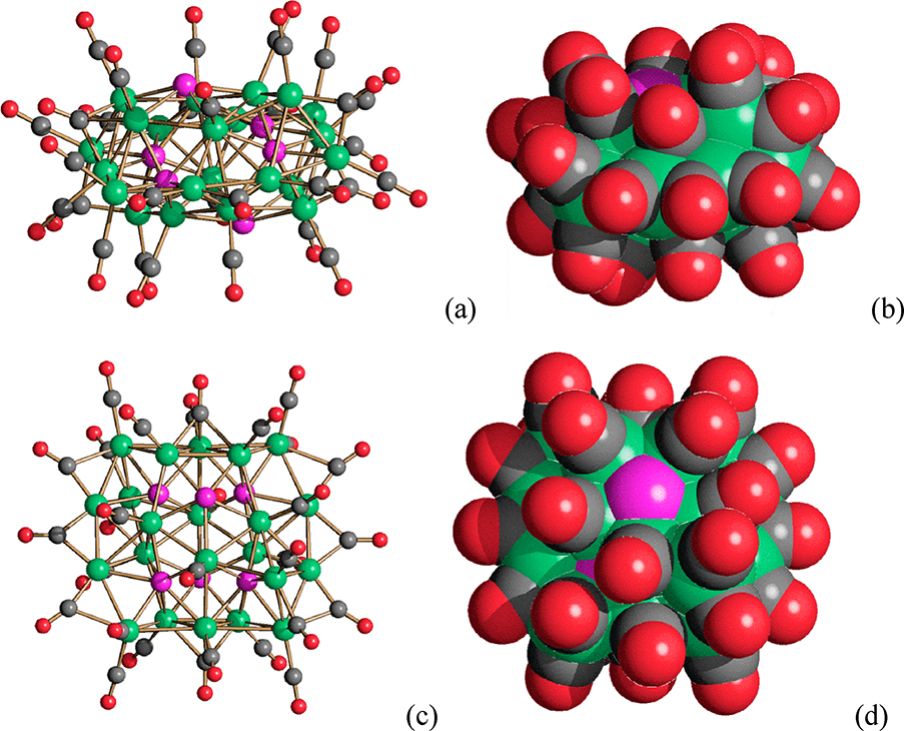
(a,c) Two views of the molecular structure of [Ni_22_P_6_(CO)_30_]^2–^ and (b,d) its space-filling model (Ni, green; P, purple; C, gray; O, red).

At the end of the reaction, Ni(CO)_4_ was eliminated under a vacuum, the Ni(II) salts washed with water, traces of [Ni_22_P_6_(CO)_30_]^2–^ extracted in thf, and eventually, [Ni_23–*x*_P_2_(CO)_30–*x*_]^4–^ (*x* = 0.82) extracted in acetone. Crystals of [NEt_4_]_4_[Ni_23–*x*_P_2_(CO)_30–*x*_]·2CH_3_COCH_3_ (*x* = 0.82) suitable for X-ray crystallography were obtained by slow diffusion of *n*-hexane on the acetone solution (Figure 3, Table 1). Crystals of the [NEt_4_]_2_[Ni_22_P_6_(CO)_30_]·2thf side product were obtained by slow diffusion of *n*-hexane on the thf solution (Figure 4, Table 1).

[Ni_23–*x*_P_2_(CO)_30–*x*_]^4–^ (*x* = 0.82) and [Ni_22_P_6_(CO)_30_]^2–^ are not stable under ESI-MS conditions (Figure S2 in Supporting Information), as often found for larger metal carbonyl clusters, especially in the presence of first-row transition metals such as Ni.

Crystals of [NEt_4_]_4_[Ni_23–*x*_P_2_(CO)_30–*x*_]·2CH_3_COCH_3_ (*x* = 0.82) display ν_CO_ at 2004(s) and 1865(ms) cm^–1^ in CH_3_CN solution. Crystals of [NEt_4_]_2_[Ni_22_P_6_(CO)_30_]·2thf display ν_CO_ at 2031(vs), 1943(w), and 1836(m) cm^–1^ in thf solution. Due to the reduced negative charge, the ν_CO_ bands of [NEt_4_]_2_[Ni_22_P_6_(CO)_30_]·2thf are considerably shifted toward higher wavenumbers compared to [NEt_4_]_4_[Ni_23–*x*_P_2_(CO)_30–*x*_]·2CH_3_COCH_3_.

**Figure 5:**
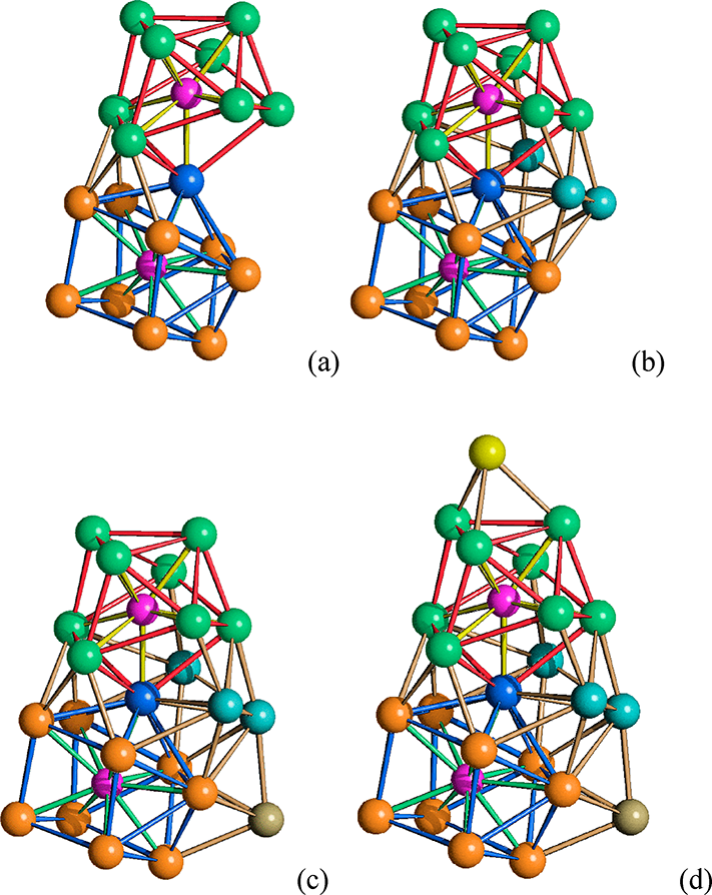
Formal building-up of the metal cage of [Ni_23–*x*_P_2_(CO)_30–*x*_]^4–^ (*x* = 0.82) (P atoms are represented in purple). (a) The Ni_18_P_2_ framework obtained by the condensation via a vertex (in blue) of a Ni_9_P monocapped square antiprism (Ni atoms in green, Ni–Ni bonds in red, Ni–P bonds in yellow) and a Ni_10_P sphenocorona (Ni atoms in orange, Ni–Ni bonds in blue, Ni–P bonds in green). (b) The Ni_21_P_2_ core of [Ni_21_P_2_(CO)_28_]^4–^ (cyan, additional Ni atoms not bonded to P). (c) The Ni_22_P_2_ core of [Ni_22_P_2_(CO)_29_]^4–^ (olive green, capping Ni with 0.68 occupancy factor). (d) The Ni_23_P_2_ core of [Ni_23_P_2_(CO)_30_]^4–^ (yellow, capping Ni with 0.50 occupancy factor).

The Ni_22_P_2_ cage present in [Ni_23–*x*_P_2_(CO)_30–*x*_]^4–^ (*x* = 0.82) is composed of one distorted Ni_9_P monocapped square antiprism (Ni atoms in green, Ni–Ni bonds in red, Ni–P bonds in yellow in Figure 5) and one distorted Ni_10_P sphenocorona (Ni atoms in orange, Ni–Ni bonds in blue, Ni–P bonds in green) fused together through a common vertex (in blue in Figure 5). This results in a Ni_18_P_2_ framework which can be completed by the addition of four further Ni atoms not bonded to any P. Three of these Ni atoms (in cyan in Figure 5) have full occupancy factors, whereas the fourth (in olive green in Figure 5) shows a refined 0.68 occupancy factor. Capping a triangular face of this Ni_22_P_2_ cage with an additional Ni atom (in yellow in Figure 5; 0.50 occupancy factor) affords the final Ni_23_P_2_ metal framework of [Ni_23_P_2_(CO)_30_]^4–^.

Thus, [Ni_23–*x*_P_2_(CO)_30–*x*_]^4–^ (*x* = 0.82) contains two Ni(CO) fragments with partial occupancy factors (0.68 and 0.50, respectively). This experimental disorder may be interpreted by two different models: (a) [Ni_23–*x*_P_2_(CO)_30–*x*_]^4–^ (*x* = 0.82) is actually a mixture of [Ni_23_P_2_(CO)_30_]^4–^ (18%) and two isomers of [Ni_22_P_2_(CO)_29_]^4–^ (82%); (b) [Ni_23–*x*_P_2_(CO)_30–*x*_]^4–^ (*x* = 0.82) is actually a mixture of [Ni_23_P_2_(CO)_30_]^4–^ (34%), two isomers of [Ni_22_P_2_(CO)_29_]^4–^ (34% and 16%, respectively), and [Ni_21_P_2_(CO)_28_]^4–^ (16%). On the basis of SC-XRD data, it is not possible to distinguish between these two models. Nonetheless, in both cases, it is possible to conclude that [Ni_23–*x*_P_2_(CO)_30–*x*_]^4–^ (*x* = 0.82) is mainly composed of [Ni_22_P_2_(CO)_29_]^4–^ (50–82%) which consists of two isomers differing in the positions of a Ni(CO) fragment. The contemporary presence of both these Ni(CO) fragments results in [Ni_23_P_2_(CO)_30_]^4–^, whereas [Ni_21_P_2_(CO)_28_]^4–^ results when they are both absent. This phenomenon is well-known for Ni carbonyl clusters, and indeed, several species differing for the addition/subtraction of a few Ni(CO) fragments have been reported.^42−44^

The cluster contains one fully interstitial Ni atom (in blue in Figure 5), 67 Ni–Ni bonding contacts (64 and 63 for the two isomers of [Ni_22_P_2_(CO)_29_]^4–^; 60 for [Ni_21_P_2_(CO)_28_]^4–^), and 19 Ni–P interactions. The interstitial Ni atom displays 11 Ni–Ni and two Ni–P contacts. The [Ni_23_P_2_(CO)_30_]^4–^ cluster is completed by 30 CO ligands, seven terminal, 19 edge bridging, and four face capping. [Ni_22_P_2_(CO)_29_]^4–^ contains 29 CO ligands: nine terminal, 16 edge bridging, and four face capping in the case of the first isomer (yellow Ni(CO) fragment, as depicted in Figure 5, is missing); seven terminal, 20 edge bridging, and two face capping in the case of the second isomer (olive green Ni(CO) fragment, as depicted in Figure 5, is missing). [Ni_21_P_2_(CO)_28_]^4–^ contains 28 CO ligands, nine terminal, 17 edge bridging, and two face capping.

On the basis of the capping principle, [Ni_23_P_2_(CO)_30_]^4–^ (304 CVE), [Ni_22_P_2_(CO)_29_]^4–^ (292 CVE), and [Ni_21_P_2_(CO)_28_]^4–^ (280 CVE) have analogous electron counts which correspond to 6*n* + 14 CMO, as found also in [Ni_14_P_2_(CO)_22_]^2–^.

**Figure 6:**
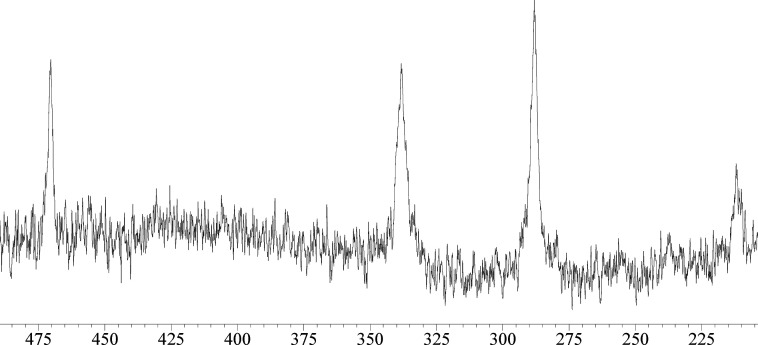
^31^P{^1^H} NMR spectrum of [Ni_23–*x*_P_2_(CO)_30–*x*_]^4–^ (*x* = 0.82) in CD_3_CN at 298 K.

The ^31^P{^1^H} NMR spectrum of [Ni_23–*x*_P_2_(CO)_30–*x*_]^4–^ (*x* = 0.82) in CD_3_CN displays four resonances at δ_P_ 470, 338, 288, and 212 ppm (Figure 6). These resonances are very broad, hampering a reliable integration of the spectrum. The weakest resonance at 212 ppm might be due to impurities or to a minor species of the [Ni_23–*x*_P_2_(CO)_30–*x*_]^4–^ (*x* = 0.82) mixture. Nonetheless, the presence of 3–4 resonances is in agreement with the fact that [Ni_23–*x*_P_2_(CO)_30–*x*_]^4–^ (*x* = 0.82) is actually a mixture of species differing for the presence and/or position of a few Ni(CO) groups (see above).

**Figure 7:**
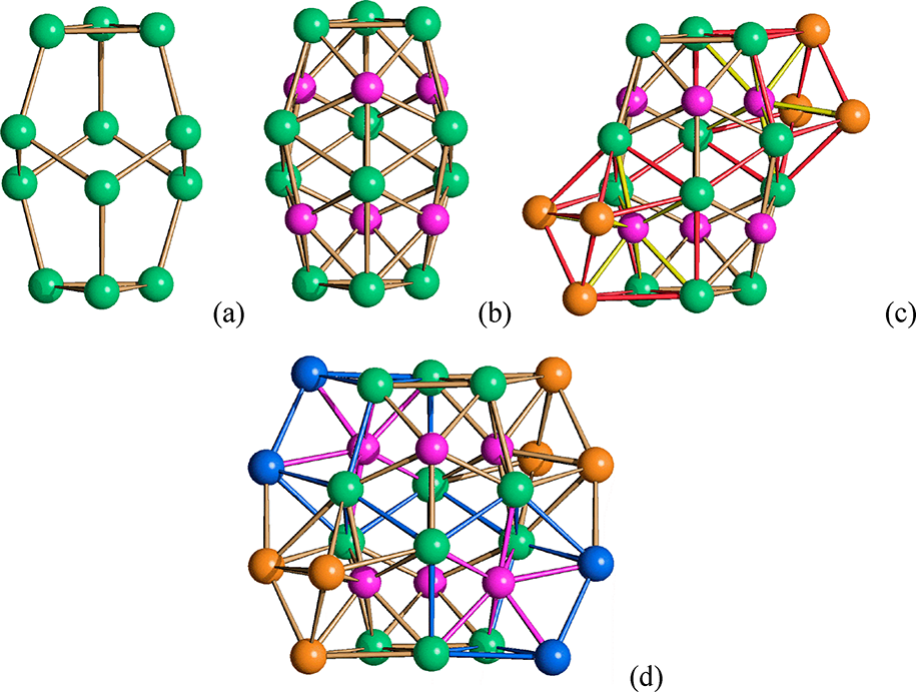
Formal building up of the metal cage of [Ni_22_P_6_(CO)_30_]^2–^. (a) The Ni_12_ polyhedron of pseudo-*D*_3*d*_ symmetry with two triangular and six pentagonal faces (green, Ni). (b) The Ni_12_P_6_ core obtained by adding six P atoms on the six pentagonal faces (purple, P). (c) The Ni_18_P_6_ framework obtained by capping two P atoms within two distorted bicapped trigonal prismatic cages (additional Ni in orange; Ni–Ni and Ni–P bonds of the bicapped trigonal prismatic cages are highlighted in red and yellow, respectively). (d) The Ni_22_P_6_ cage obtained by partially capping two P atoms within two distorted monocapped trigonal prismatic cages (additional Ni in blue; Ni–Ni and Ni–P bonds of the monocapped trigonal prismatic cages are highlighted in blue and purple, respectively).

Two views of the molecular structure of [Ni_22_P_6_(CO)_30_]^2–^ are reported in Figure 4, whereas the formal building up of its Ni_22_P_6_ cage is represented in Figure 7. The core of the cluster consists of a Ni_12_ polyhedron of *pseudo D*_3*d*_ symmetry, which possesses two parallel triangular and six adjacent pentagonal faces. The six P atoms are capping the six pentagonal faces, resulting in a Ni_12_P_6_ cage. The environments of these six P atoms are rather different: (a) Two P atoms are exposed on the cluster surface, being connected only to the five Ni atoms of the pentagonal face. (b) Two P atoms are fully interstitial, being encapsulated within distorted Ni_8_P bicapped trigonal prismatic cages, obtained by adding three further Ni atoms per P atom (in orange in Figure 7). (c) Two P atoms are in semi-interstitial positions within highly distorted Ni_7_P monocapped trigonal prismatic cages, resulting from the addition of two further Ni atoms per P atom (in blue in Figure 7).

The [Ni_22_P_6_(CO)_30_]^2–^ cluster possesses 30 CO ligands, 22 terminal, six edge bridging, and two face capping. The cluster displays 312 CVEs, corresponding to 6*n* + 24 CMOs, by considering all six P atoms as contributing with five electrons each. Conversely, by considering the two exposed P atoms contributing with only three electrons to the electron count, the cluster possesses 308 CVEs and 6*n* + 22 CMOs. In both cases, [Ni_22_P_6_(CO)_30_]^2–^ is rather electron rich, as often found in larger Ni carbonyl clusters containing several interstitial heteroatoms.^45−47^ Indeed, the electron count of Ni–P carbonyl clusters seems to increase considerably by increasing the number of P atoms, that is, [Ni_11_P(CO)_18_]^3–^ (6*n* + 11), [Ni_14_P_2_(CO)_22_]^2–^ (6*n* + 14), [Ni_23–*x*_P_2_(CO)_29–*x*_]^4–^ (6*n* + 14), [Ni_39_P_3_(CO)_44_]^6–^ (6*n* + 16), [HNi_31_P_4_(CO)_34_]^5–^ (6*n* + 21), and [Ni_22_P_6_(CO)_30_]^2–^ (6*n* + 22 or 6*n* + 24).

In agreement with the solid state structure, the ^31^P{^1^H} NMR spectrum of [Ni_22_P_6_(CO)_30_]^2–^ in CD_3_CN displays three equally intense resonances at δ_P_ 503.2, 401.4, and 383.6 ppm (Figure S3 in Supporting Information). These resonances show some fine structures likely due to a larger and a smaller coupling constant, that is *J*_PP_ = 90 and 30 Hz, respectively.

### Synthesis and Molecular Structure of [Ni_22–*x*_P_2_(CO)_29–*x*_]^4–^ (*x* = 0.84)

**Figure 8:**
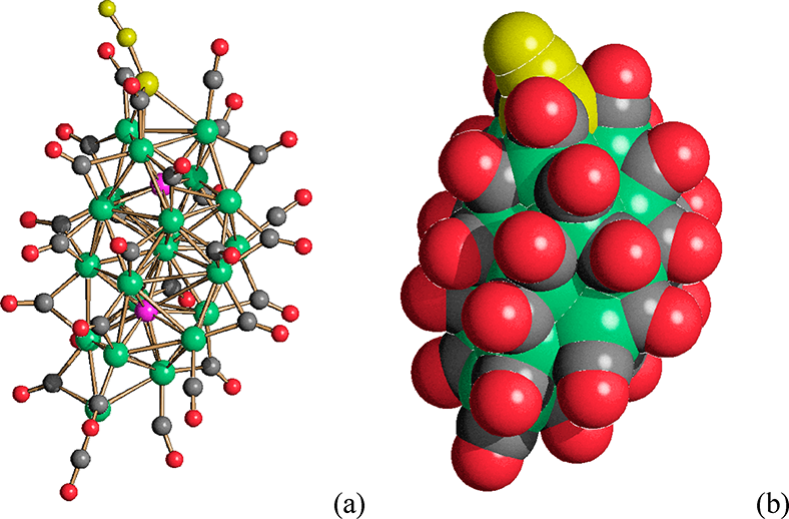
(a) The molecular structure of [Ni_22–*x*_P_2_(CO)_29–*x*_]^4–^ (*x* = 0.84) and (b) its space-filling model (Ni, green; P, purple; C, gray; O, red). The Ni(CO) fragment with a partial occupancy factor is represented in yellow.

[Ni_22–*x*_P_2_(CO)_29–*x*_]^4–^ (*x* = 0.84) resulted from the reaction of [NEt_4_]_2_[Ni_6_(CO)_12_] with 0.4–0.5 equiv of POCl_3_ in CH_3_CN. At the end of the reaction, Ni(CO)_4_ was removed in a vacuum. The Ni(II) salts were washed with H_2_O. Traces of [Ni_6_(CO)_12_]^2–^ were extracted in thf, and eventually, [Ni_22–*x*_P_2_(CO)_29–*x*_]^4–^ (*x* = 0.84) was extracted in acetone. Crystals of [NEt_4_]_4_[Ni_22–*x*_P_2_(CO)_29–*x*_]·2CH_3_COCH_3_ (*x* = 0.84) suitable for SC-XRD were obtained by slow diffusion of *n*-hexane on the acetone solutions (Figure 8, Table 1).

Crystals of [NEt_4_]_4_[Ni_22–*x*_P_2_(CO)_29–*x*_]·2CH_3_COCH_3_ (*x* = 0.84) display ν_CO_ at 2003(vs), 1962(sh), and 1869(s) cm^–1^ in CH_3_CN. The crystals actually contain a mixture of [Ni_22_P_2_(CO)_29_]^4–^ (16%) and [Ni_21_P_2_(CO)_28_]^4–^ (84%), since there is a Ni(CO) fragment with 0.16 refined occupancy factor (in yellow in Figure 8). In agreement with the presence of a mixture of two products (a major one and a minor one), the ^31^P{^1^H} NMR spectrum shows two broad resonances at δ_P_ 212.9 and 163.8 ppm (Figure S4 in Supporting Information). As in the case of [Ni_23–*x*_P_2_(CO)_30–*x*_]^4–^ (*x* = 0.82), [Ni_22–*x*_P_2_(CO)_29–*x*_]^4–^ (*x* = 0.84) was not stable under ESI-MS conditions.

Despite the fact that [Ni_22–*x*_P_2_(CO)_29–*x*_]^4–^ (*x* = 0.84) and [Ni_23–*x*_P_2_(CO)_30–*x*_]^4–^ (*x* = 0.82) have very similar formulas, they display significantly different metal cages, which might be viewed as isomers. Structural isomerism in molecular clusters of increasing sizes is rather interesting in view of its relevance to the field of metal nanoclusters, nanoparticles, and nanomaterials.^48−52^

**Figure 9:**
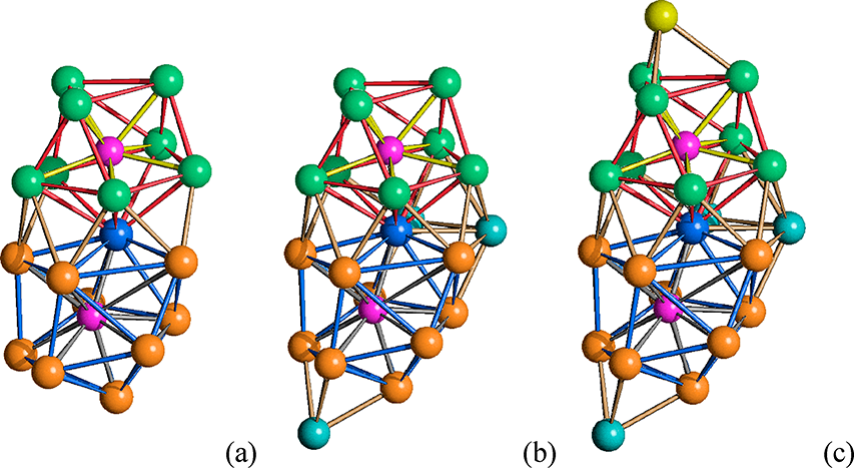
Formal building-up of the metal cage of [Ni_22–*x*_P_2_(CO)_29–*x*_]^4–^ (*x* = 0.84) (P atoms are represented in purple). (a) The Ni_18_P_2_ framework obtained by the condensation via a vertex (in blue) of a Ni_9_P monocapped square antiprism (Ni atoms in green, Ni–Ni bonds in red, Ni–P bonds in yellow) and a Ni_10_P bicapped square antiprism (Ni atoms in orange, Ni–Ni bonds in blue, Ni–P bonds in gray). (b) The Ni_21_P_2_ core of [Ni_21_P_2_(CO)_28_]^4–^ (cyan, additional Ni atoms not bonded to P). (c) The Ni_22_P_2_ core of [Ni_22_P_2_(CO)_29_]^4–^ (yellow, capping Ni with partial occupancy factor).

The main difference between [Ni_22–*x*_P_2_(CO)_29–*x*_]^4–^ (*x* = 0.84) and [Ni_23–*x*_P_2_(CO)_30–*x*_]^4–^ (*x* = 0.82) consists of the fact that the former results from one distorted Ni_9_P monocapped square antiprism, as [Ni_23–*x*_P_2_(CO)_30–*x*_]^4–^ (*x* = 0.5), and one distorted Ni_10_P bicapped square antiprism, rather than a Ni_10_P sphenocorona. These two cages are fused together through a common vertex (Figure 9), resulting in a Ni_18_P_2_ framework which may be viewed as an isomer of the Ni_18_P_2_ framework present in [Ni_23–*x*_P_2_(CO)_30–*x*_]^4–^ (*x* = 0.82). The Ni_21_P_2_ cage of [Ni_22–*x*_P_2_(CO)_29–*x*_]^4–^ (*x* = 0.84) is completed by the addition of three further Ni atoms not bonded to any P. Capping a triangular face of this with an additional Ni atoms affords the final Ni_22_P_2_ metal framework of [Ni_22_P_2_(CO)_29_]^4–^.

The cluster contains one fully interstitial Ni atom (in blue in Figure 9), 63 Ni–Ni bonding contacts (60 for [Ni_21_P_2_(CO)_28_]^4–^), and 19 Ni–P interactions. The interstitial Ni atom displays 12 Ni–Ni and two Ni–P contacts. The [Ni_22_P_2_(CO)_29_]^4–^ cluster is completed by 29 CO ligands, four terminal and 25 edge bridging. Conversely, [Ni_21_P_2_(CO)_28_]^4–^ contains 28 CO ligands, six terminal and 22 edge bridging.

The electron count of the [Ni_22_P_2_(CO)_29_]^4–^ (292 CVE) and [Ni_21_P_2_(CO)_28_]^4–^ (280 CVE) clusters found in [Ni_22–*x*_P_2_(CO)_29–*x*_]^4–^ (*x* = 0.84) corresponds to 6*n* + 14 CMO, as in the case of [Ni_23–*x*_P_2_(CO)_30–*x*_]^4–^ (*x* = 0.82).

### Synthesis and Molecular Structure of [Ni_39_P_3_(CO)_44_]^6–^

**Figure 10:**
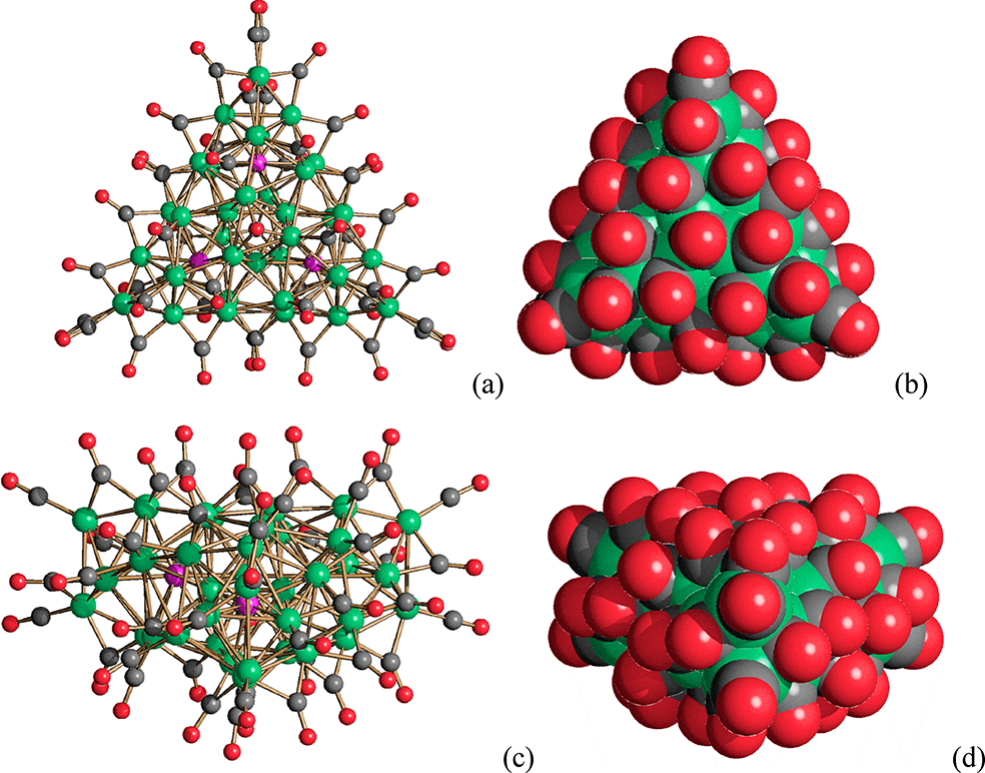
(a,c) The molecular structure of [Ni_39_P_3_(CO)_44_]^6–^ and (b,d) its space-filling model (Ni, green; P, purple; C, gray; O, red).

[Ni_39_P_3_(CO)_44_]^6–^ was obtained following a very similar procedure to that described for the synthesis of [Ni_22–*x*_P_2_(CO)_29–*x*_]^4–^ (*x* = 0.84) but performing the reaction in thf rather than CH_3_CN. Thus, [NEt_4_]_2_[Ni_6_(CO)_12_] was reacted with 0.4–0.5 equiv of POCl_3_ in thf, and after workup, [Ni_39_P_3_(CO)_44_]^6–^ was extracted in CH_3_CN. Slow diffusion on *n*-hexane and di-iso-propyl-ether afforded a few crystals of [NEt_4_]_6_[Ni_39_P_3_(CO)_44_]·C_6_H_14_·solv suitable for SC-XRD (Figure 10 and Table 1). The compound displays ν_CO_ at 1998(vs) and 1868(s) cm^–1^ in CH_3_CN.

**Figure 11:**
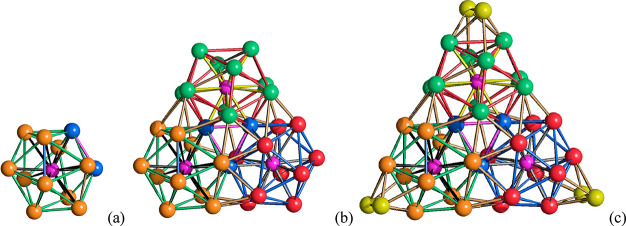
Formal building-up of the metal cage of [Ni_39_P_3_(CO)_44_]^6–^ (P atoms are represented in purple). (a) One of the three P-centered Ni_12_P icosahedra (Ni atoms belonging only to this icosahedron in orange, Ni atoms shared with other icosahedra in blue, Ni–Ni bonds in green, Ni–P bonds in black). (b) The Ni_33_P_3_ core obtained by fusing three Ni_12_P icosahedra sharing three atoms (different colors have been used for the Ni atoms, Ni–Ni and Ni–P bonds within each icosahedron; the shared Ni_3_ triangle is represented in blue, its Ni–Ni bonds in purple). (c) The Ni_39_P_3_ core of [Ni_39_P_3_(CO)_44_]^6–^ obtained after the addition of three Ni_2_ units, one per each icosahedron (yellow, additional Ni atoms not bonded to P).

The structure of [Ni_39_P_3_(CO)_44_]^6–^ is based on a Ni_39_P_3_ metal core of idealized *D*_3*h*_ symmetry and 39 CO ligands, six terminal, 36 edge bridging, and two face capping (Figure 11). The metal core of the cluster is composed of three Ni_12_P centered icosahedra fused together around a 3-fold axis. Each Ni_12_P centered icosahedron shares two contiguous Ni atoms with the other two icosahedra, resulting in a Ni_33_P_3_ framework. The three Ni atoms shared by the three icosahedra form a fully interstitial Ni_3_ triangle. Each Ni atom within this triangle is bonded to 10 Ni atoms and two P atoms. The Ni_39_P_3_ core of the cluster is completed by adding three Ni_2_ units, one per Ni_12_P icosahedron. These two additional Ni atoms are capping two adjacent triangular faces within each icosahedron. The additional Ni atoms are not bonded to P, and they are coordinated to the six terminal carbonyls present in the cluster.

This represents the first case of a phosphide atom enclosed within an icosahedral cage in a transition metal cluster. Indeed, P atoms are usually found in smaller cages with coordination numbers comprised in the range 5–10.^22−30,40,41,53−56^ Icosahedra are often found with larger heteroatoms, such as Sn, Sb, Bi, and Ge.^57,58^

## Conclusions

Five new molecular nickel phosphide carbonyl clusters, that is, [Ni_14_P_2_(CO)_22_]^2–^, [Ni_22–*x*_P_2_(CO)_29–*x*_]^4–^ (*x* = 0.84), [Ni_39_P_3_(CO)_44_]^6–^, [Ni_23–*x*_P_2_(CO)_30–*x*_]^4–^ (*x* = 0.82), and [Ni_22_P_6_(CO)_30_]^2–^, have been structurally characterized, and they add to the previously reported [Ni_11_P(CO)_18_]^3–^ and [H_6–*n*_Ni_31_P_4_(CO)_39_]^*n*−^ (*n* = 4, 5). The sizes of the metal cores of these clusters range from 0.59 to 1.10 nm, and their overall dimensions including the CO ligands are 1.16–1.63 nm (Table 2). Thus, the sizes of these molecular clusters are comparable to those of ultrasmall metal nanoparticles, molecular nanoclusters, or atomically precise metal nanoparticles.^59−61^ In this respect, interstitial phosphide atoms seem to be as effective as carbides in order to stabilize molecular nickel carbonyl nanoclusters.^42−45,59^

**Table 2:** Dimensions of the Known Ni–P–CO Molecular Clusters

	metal core	size including CO
[Ni_11_P(CO)_18_]^3–^	0.59 nm	1.16 nm
[Ni_14_P_2_(CO)_22_]^2–^	0.72 nm	1.23 nm
[Ni_22_P_6_(CO)_30_]^2–^	0.99 nm	1.40 nm
[Ni_23–*x*_P_2_(CO)_30–*x*_]^4–^ (*x* = 0.82)	0.98 nm	1.54 nm
[Ni_22–*x*_P_2_(CO)_29–*x*_]^4–^ (*x* = 0.84)	1.08 nm	1.59 nm
[H_6–*n*_Ni_31_P_4_(CO)_39_]^*n*−^ (*n* = 4, 5)	1.04 nm	1.60 nm
[Ni_39_P_3_(CO)_44_]^6–^	1.10 nm	1.63 nm

**Figure 12:**
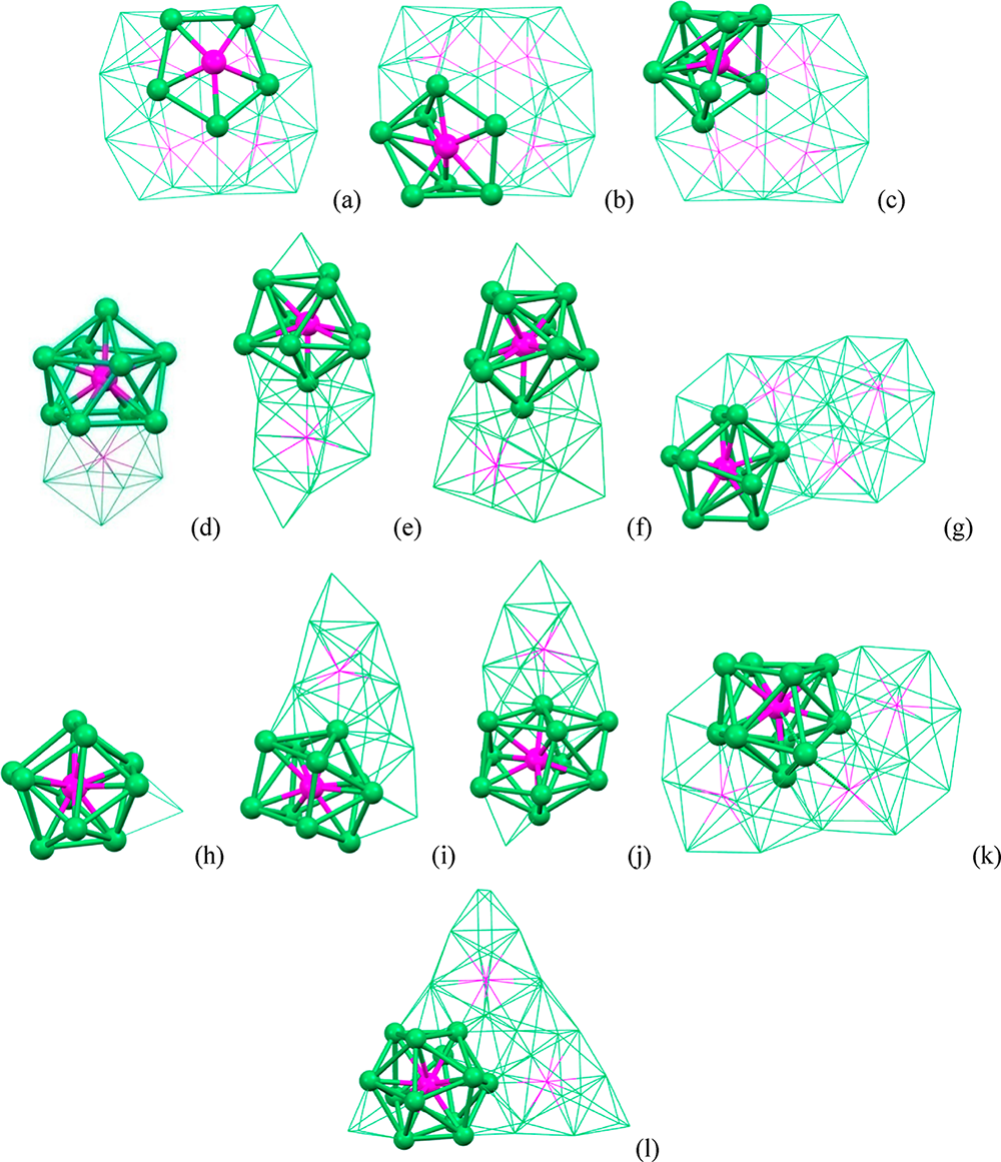
Diverse environments of P atoms in Ni–P–CO clusters. Ni_5_P, pentagonal pyramid of (a) [Ni_22_P_6_(CO)_30_]^2–^; Ni_7_P, monocapped trigonal prism of (b) [Ni_22_P_6_(CO)_30_]^2–^; Ni_8_P, bicapped trigonal prism of (c) [Ni_22_P_6_(CO)_30_]^2–^; Ni_9_P, monocapped square antiprism of (d) [Ni_14_P_2_(CO)_22_]^2–^, (e) [Ni_22–*x*_P_2_(CO)_29–*x*_]^4–^, (f) [Ni_23–*x*_P_2_(CO)_30–*x*_]^4–^, and (g) [H_6–*n*_Ni_31_P_4_(CO)_39_]^*n*−^ (*n* = 4, 5); Ni_10_P, sphenocorona of (h) [Ni_11_P(CO)_18_]^3–^ and (i) [Ni_23–*x*_P_2_(CO)_30–*x*_]^4–^; Ni_10_P, bicapped square antiprism of (j) [Ni_22–*x*_P_2_(CO)_29–*x*_]^4–^ and (k) [H_6–*n*_Ni_31_P_4_(CO)_39_]^*n*−^ (*n* = 4, 5); Ni_12_P, icosahedron of (l) [Ni_39_P_3_(CO)_44_]^6–^.

The environment of the P atoms within these molecular Ni–P–CO nanoclusters displays a rich diversity. Indeed, they may be fully interstitial, semiexposed, or exposed in very diverse cages, that is, Ni_5_P pentagonal pyramid (exposed P in a pentagonal face), (highly distorted) Ni_7_P monocapped trigonal prism (semiexposed P), Ni_8_P bicapped trigonal prism, Ni_9_P monocapped square antiprism, Ni_10_P sphenocorona, Ni_10_P bicapped square antiprism, and Ni_12_P icosahedron (Figure 12).

This structural diversity is paralleled by the richness of structural motives found in Ni–P phases and nanostructures. Indeed, Ni-rich phases show isolated P atoms inside a Ni_9_P tricapped trigonal prismatic cage (Ni_2_P phase), a distorted Ni_9_P monocapped square antiprismatic cage (Ni_3_P phase), and a mixture of Ni_10_P sphenocorona and Ni_9_P monocapped cubic cages (Ni_12_P_5_ phase). Therefore, some of these structural motives are common to molecular metal carbonyl clusters and solid state Ni–P phases (Ni_9_P monocapped square antiprism, Ni_10_P sphenocorona); some others have been found for the moment only in metal carbonyl clusters (Ni_5_P pentagonal pyramid, Ni_7_P monocapped trigonal prism, Ni_8_P bicapped trigonal prism, Ni_10_P bicapped square antiprism, Ni_12_P icosahedron) or solely in solid state Ni–P phases (Ni_9_P tricapped trigonal prism, Ni_9_P monocapped cube). In addition, P-richer solid state Ni–P phases display direct P–P bonds which may result in P_2_ units (NiP and the high-pressure cubic NiP_2_), zig-zig chains (monoclinic NiP_2_), and P_4_ units (NiP_3_), as well as more complex and less regular structures. In contrast, molecular Ni–P carbonyl clusters containing direct P–P interactions have not been isolated yet.

## Supporting Information Available

NMR spectra, crystals and experimental details, ORTEP drawings of all the structures (PDF)

## Accession Codes

CCDC 2025497, 2025498, 2025499, 2025500, 2025501, and 2025502 contain the supplementary crystallographic data for this paper. These data can be obtained free of charge via www.ccdc.cam.ac.uk/data_request/cif, or by emailing data_request@ccdc.cam.ac.uk, or by contacting The Cambridge Crystallographic Data Centre, 12, Union Road, Cambridge CB2 1EZ, UK; fax: + 44 1223 336033.
