# Supplementary material for: Structural Diversity in Molecular Nickel Phosphide Carbonyl Nanoclusters
Source: Inorg Chem. 2020 Oct 21;59(21):16016–26. doi: 10.1021/acs.inorgchem.0c02572 (PMC8015230; doi:10.1021/acs.inorgchem.0c02572)

Supporting Information for

Structural Diversity in Molecular Nickel Phosphide Carbonyl Nanoclusters

Chiara Capacci, Cristiana Cesari, Cristina Femoni, Maria Carmela Iapalucci, Federica Mancini, Silvia Ruggieri and Stefano Zacchini*

Dipartimento di Chimica Industriale "Toso Montanari", Università di Bologna, Viale Risorgimento 4 - 40136 Bologna. Italy.

	Page/s
$^{31}P\{^1H\}$ NMR spectrum of $[Ni_{14}P_2(CO)_{22}]^{2-}$	S2
ESI-MS spectrum of $[NEt_4]_4[Ni_{23-x}P_2(CO)_{30-x}]\cdot 2CH_3COCH_3$ (x = 0.82)	S3
$^{31}P\{^1H\}$ NMR spectrum of $[Ni_{22}P_6(CO)_{30}]^{2-}$	S4
$^{31}P\{^1H\}$ NMR spectrum of $[Ni_{22-x}P_2(CO)_{29-x}]^{4-}$ (x = 0.84)	S5
Crystal data and experimental details	S6-S9
ORTEP drawings of the structures	S10-S14

Figure S1

$^{31}P\{^1H\}$ NMR spectrum of $[Ni_{14}P_2(CO)_{22}]^{2-}$ in CD_2Cl_2 at 298 K.

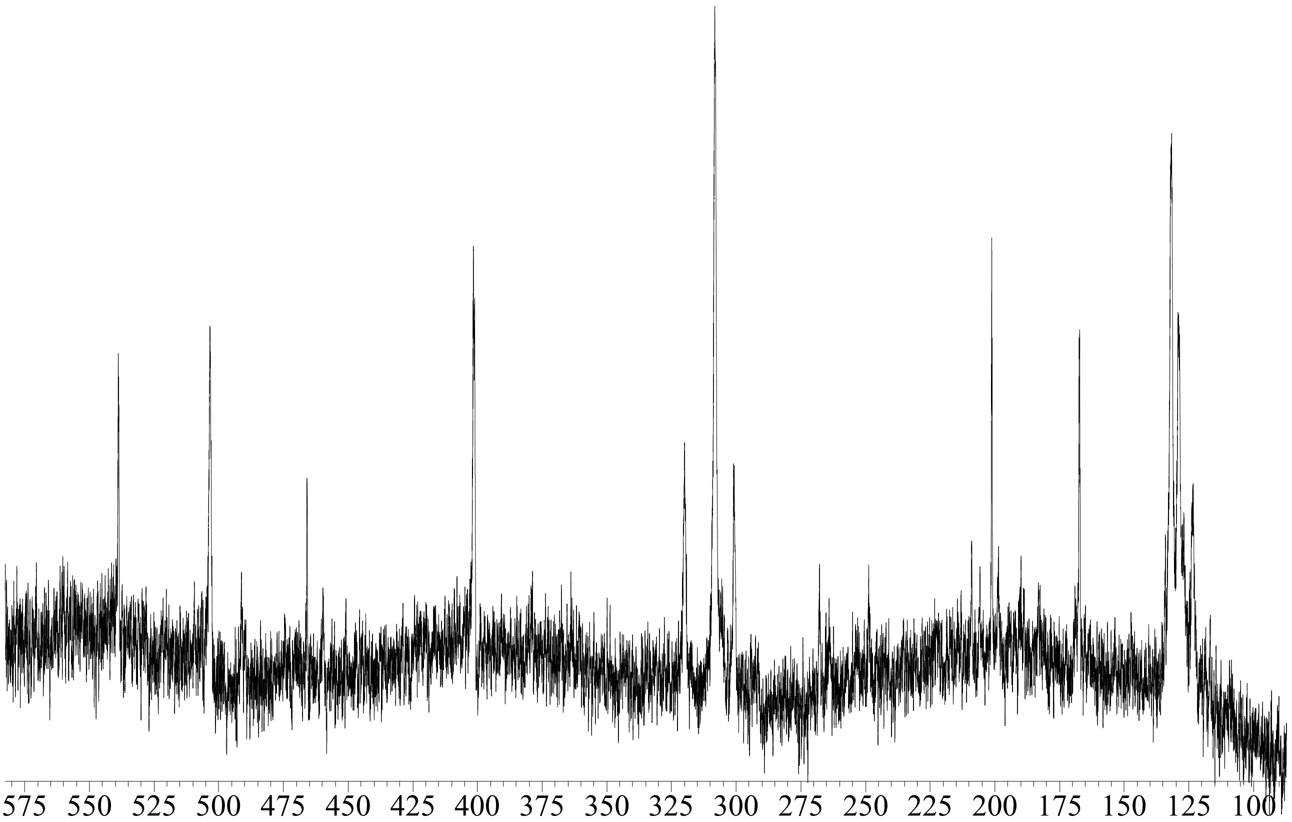

Figure S2

ESI-MS spectrum in CH_3CN (ES–) of $[NEt_4]_4[Ni_{23-x}P_2(CO)_{30-x}]\cdot 2CH_3COCH_3$ (x = 0.82).

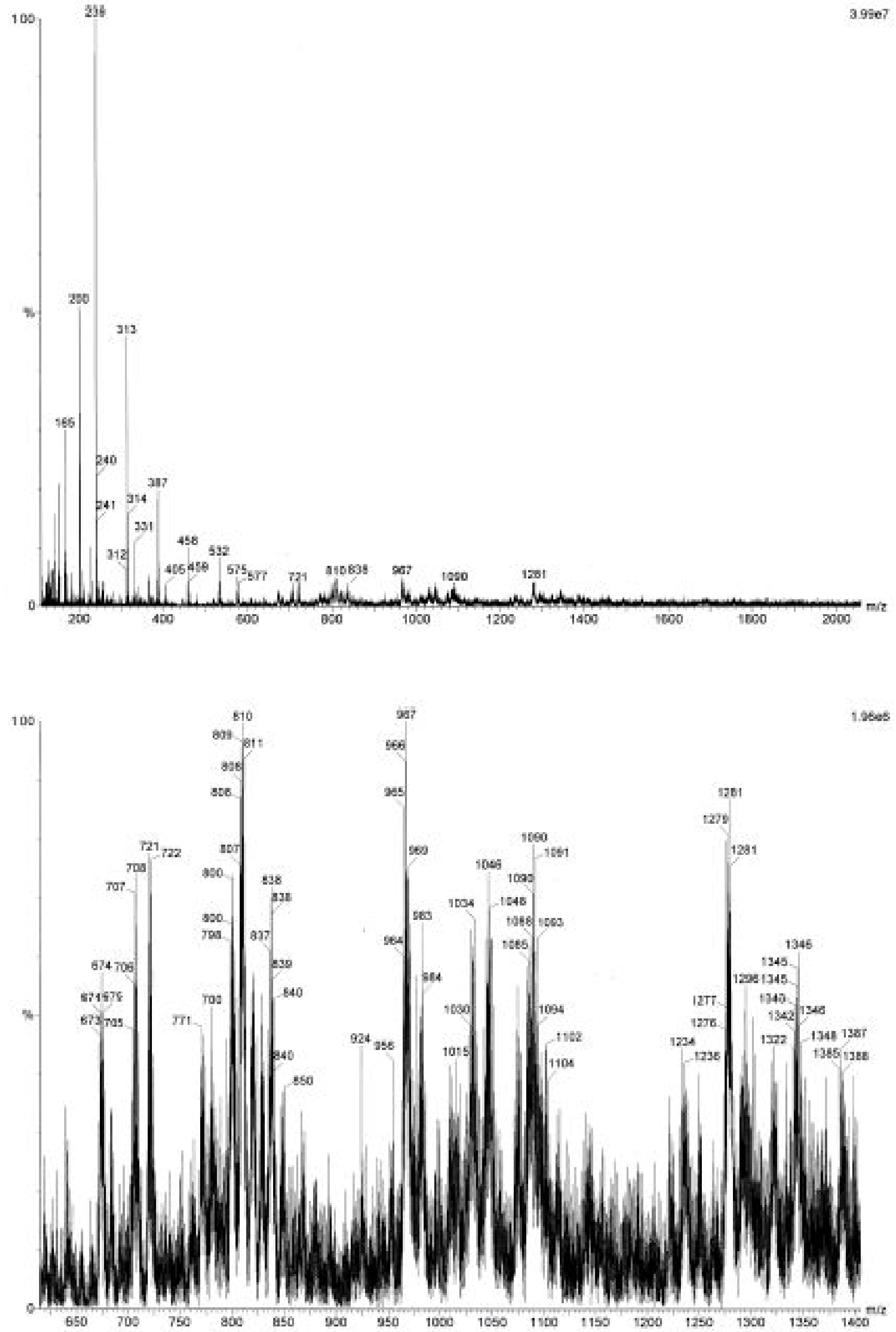

Figure S3

$^{31}P\{^{1}H\}$ NMR spectrum of $[Ni_{22}P_6(CO)_{30}]^{2-}$ in CD_3CN at 298 K.

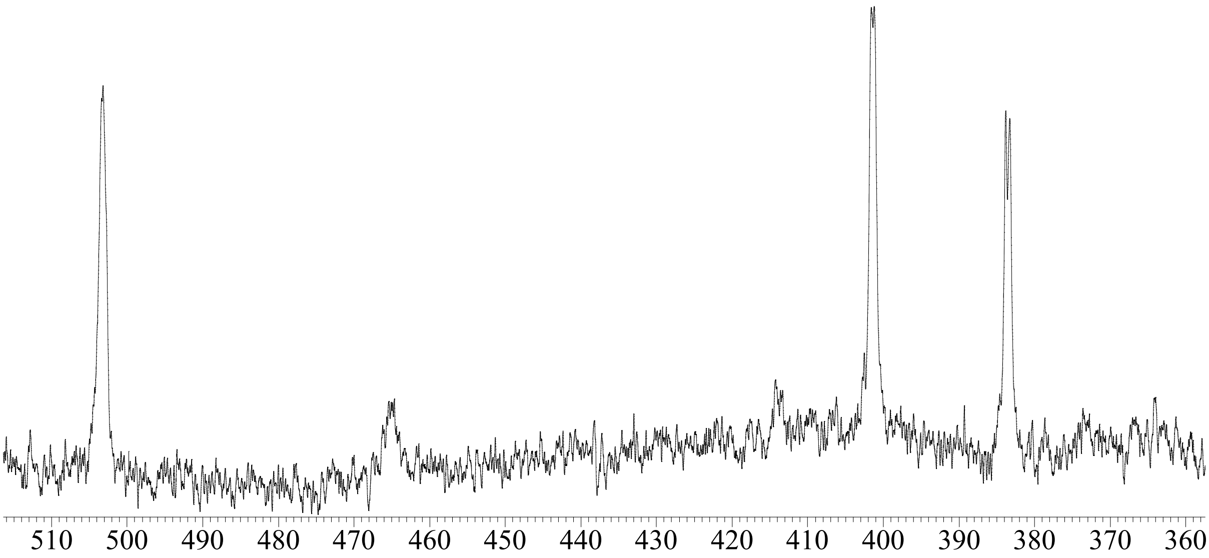

Figure S4

$^{31}P\{^1H\}$ NMR spectrum of $[Ni_{22-x}P_2(CO)_{29-x}]^{4-}$ (x = 0.84) in CD_3CN at 298 K. The sharp resonance at ca. 200 ppm is due to a hydrolysis product of $POCl_3$.

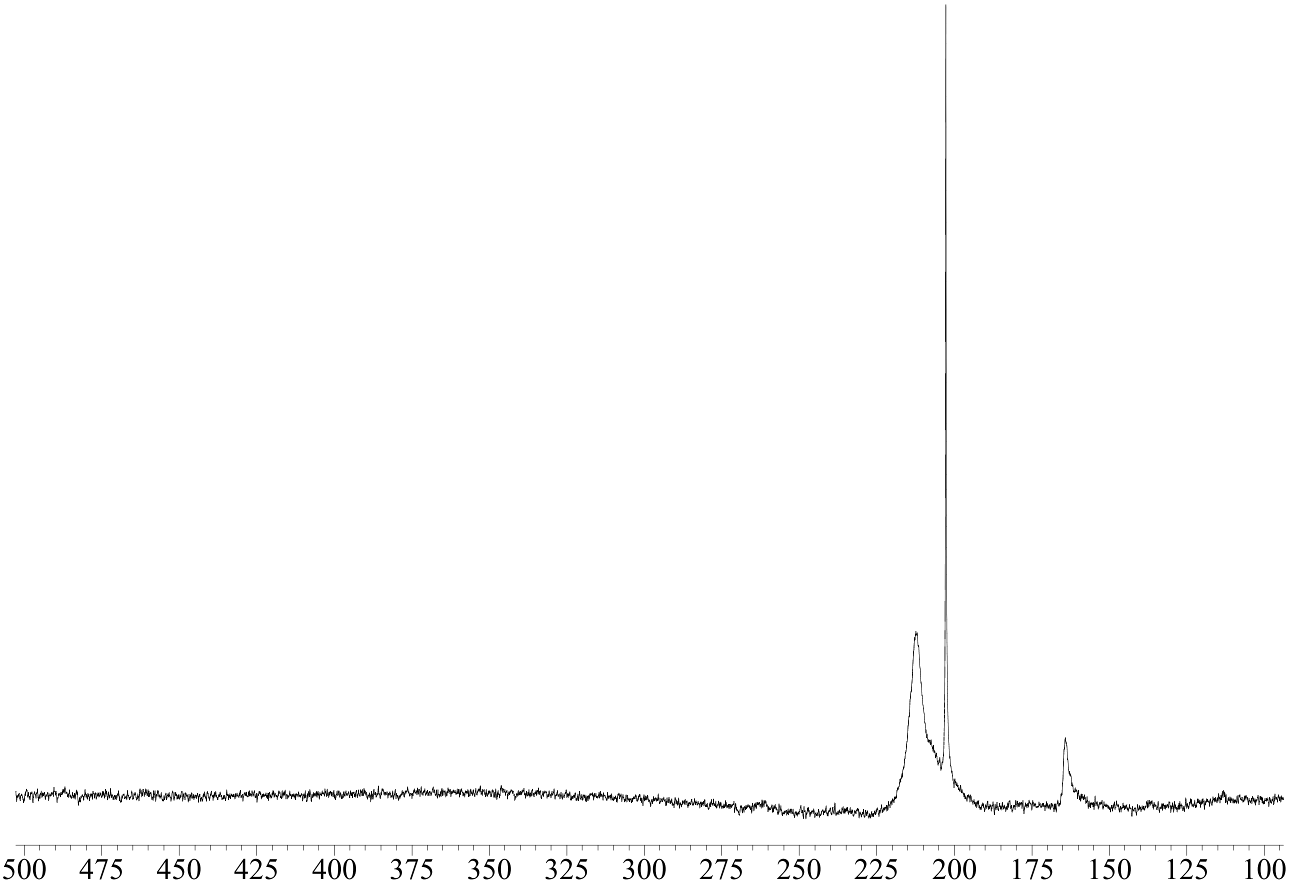

Table S1

Crystal data and experimental details for $[NBu_4]_2[Ni_{14}P_2(CO)_{22}]$ ($P2_1/n$), $[NBu_4]_2[Ni_{14}P_2(CO)_{22}]$ ($C2/c$), $[NEt_4]_4[Ni_{23-x}P_2(CO)_{30-x}]\cdot CH_3COCH_3\cdot solv$ (x = 0.82), $[NEt_4]_4[Ni_{22-x}P_2(CO)_{29-x}]\cdot 2CH_3COCH_3$(x = 0.84), $[NEt_4]_2[Ni_{22}P_6(CO)_{30}]\cdot 2thf$ and $[NEt_4]_6[Ni_{39}P_3(CO)_{44}]\cdot C_6H_{14}\cdot solv$.

	**$[NBu_4]_2[Ni_{14}P_2(CO)_{22}]$ ($P2_1/n$)**	**$[NBu_4]_2[Ni_{14}P_2(CO)_{22}]$ ($C2/c$)**	**$[NEt_4]_4[Ni_{23-x}P_2(CO)_{30-x}]\cdot CH_3COCH_3\cdot solv$ (x = 0.82)**
Formula	$C_{54}H_{72}N_2Ni_{14}O_{22}P_2$	$C_{54}H_{72}N_2Ni_{14}O_{22}P_2$	$C_{64.18}H_{86}N_4Ni_{22.18}O_{30.18}P_2$
Fw	1985.02	1985.02	2760.97
T, K	294(2)	295(2)	100(2)
λ, Å	0.71073	0.71073	0.71073
Crystal system	Monoclinic	Monoclinic	Monoclinic
Space Group	$P2_1/n$	$C2/c$	$P2_1/n$
a, Å	11.2393(9)	24.397(3)	14.9034(18)
b, Å	20.1941(15)	14.5786(19)	23.394(3)
c, Å	16.8025(13)	23.548(5)	26.186(3)
β, °	100.4700(10)	117.619(2)	99.202(2)
Cell Volume, Å^3	3750.1(5)	7421(2)	9012.3(19)
Z	2	4	4
D_c, g cm^{-3}	1.758	1.777	2.035
μ, mm^{-1}	3.532	3.569	4.618
F(000)	2016	4032	5567
Crystal size, mm	0.23×0.18×0.13	0.20×0.13×0.11	0.16×0.13×0.11
θ limits, °	1.59–27.00	1.68–27.00	1.479–25.00
Index ranges	$-14 \le h \le 14$<br>$-25 \le k \le 25$<br>$-21 \le l \le 21$	$-31 \le h \le 31$<br>$-18 \le k \le 18$<br>$-30 \le l \le 30$	$-17 \le h \le 17$<br>$-27 \le k \le 27$<br>$-31 \le l \le 31$
Reflections collected	41265	40801	83901
Independent reflections	8186 [R_{int} = 0.0286]	8100 [R_{int} = 0.0819]	15839 [R_{int} = 0.0858]
Completeness to θ max	99.9%	100.0%	99.8%
Data / restraints / parameters	8186 / 191 / 424	8100 / 214 / 424	15839 / 1123 / 1336
Goodness on fit on F^2	1.026	1.013	1.005
R_1 (I > 2σ(I))	0.0436	0.0653	0.0403
wR_2 (all data)	0.1416	0.2346	0.0969
Largest diff. peak and hole, e Å^{-3}	0.922 / –0.572	1.832 / –0.610	0.758 / –0.574

	**$[NEt_4]_4[Ni_{22-x}P_2(CO)_{29-x}]\cdot 2CH_3COCH_3$ (x = 0.84)**	**$[NEt_4]_2[Ni_{22}P_6(CO)_{30}]\cdot 2thf$**	**$[NEt_4]_6[Ni_{39}P_3(CO)_{44}]\cdot C_6H_{14}\cdot solv$**
Formula	$C_{66.16}H_{92}N_4Ni_{21.16}O_{30.16}P_2$	$C_{54}H_{56}N_2Ni_{22}O_{32}P_6$	$C_{98}H_{134}N_6Ni_{39}O_{44}P_3$
Fw	2730.37	2722.44	4482.70
T, K	100(2)	100(2)	100(2)
λ, Å	0.71073	0.71073	0.71073
Crystal system	Monoclinic	Triclinic	Hexagonal
Space Group	$P2_1/n$	$P\bar{1}$	$P6_322$
a, Å	15.2015(13)	11.1607(14)	25.320(2)
b, Å	26.212(2)	13.4566(17)	25.320(2)
c, Å	22.4308(18)	15.1954(19)	15.5072(12)
□, °	90	114.343(3)	90
β, °	100.023(3)	99.678(3)	90
□, °	90	94.596(3)	120
Cell Volume, $Å^3$	8801.3(13)	2021.1(4)	8609.8(15)
Z	4	1	2
D_c, g cm^{-3}	2.061	2.237	1.729
μ, mm^{-1}	4.518	5.182	4.233
F(000)	5523	1356	4506
Crystal size, mm	0.18×0.16×0.14	0.25×0.23×0.15	0.18×0.16×0.12
θ limits, °	1.554–26.000	1.508–25.099	1.608–25.097
Index ranges	$-18 \le h \le 16$<br>$-32 \le k \le 32$<br>$-27 \le l \le 27$	$-13 \le h \le 13$<br>$-16 \le k \le 16$<br>$-18 \le l \le 18$	$-30 \le h \le 30$<br>$-30 \le k \le 30$<br>$-18 \le l \le 18$
Reflections collected	96884	29352	79464
Independent reflections	17053 [R_{int} = 0.1261]	7126 [R_{int} = 0.0852]	5135 [R_{int} = 0.1371]
Completeness to θ max	99.0%	99.1%	99.8%
Data / restraints / parameters	17053 / 340 / 1069	7126 / 186 / 543	5135 / 86 / 298
Goodness on fit on F^2	1.100	1.131	1.049
R_1 (I > 2σ(I))	0.1033	0.1000	0.0454
wR_2 (all data)	0.2900	0.2368	0.1231
Largest diff. peak and hole, e $Å^{-3}$	2.424 / –1.681	2.821 / –1.550	0.651 / –0.939

$[NBu_4]_2[Ni_{14}P_2(CO)_{22}]$ ($P2_1/n$): The asymmetric unit of the unit cell contains half of a cluster anion (located on an inversion centre) and one $[NBu_4]^+$ cation (located on a general positions). Similar *U* parameter restraints have been applied to the C, N and O atoms (SIMU line in SHELXL, s.u. 0.01). Restraints to bond distances were applied as follow (s.u. 0.005): 1.47 Å for C–N and 1.53 Å for C–C in $[NBu_4]^+$.

$[NBu_4]_2[Ni_{14}P_2(CO)_{22}]$ ($C2/c$): The asymmetric unit of the unit cell contains half of a cluster anion (located on an inversion centre) and one $[NBu_4]^+$ cation (located on a general positions). Similar *U* parameter restraints have been applied to the C, N and O atoms (SIMU line in SHELXL, s.u. 0.005). Restraints to bond distances were applied as follow (s.u. 0.01): 1.47 Å for C–N and 1.53 Å for C–C in $[NBu_4]^+$.

$[NEt_4]_4[Ni_{23-x}P_2(CO)_{30-x}]\cdot CH_3COCH_3\cdot solv$ ($x = 0.82$): The asymmetric unit of the unit cell contains one cluster anion, four $[NEt_4]^+$ cations and one CH_3COCH_3 molecule all located on a general positions. The unit cell contains an additional total potential solvent accessible void of 318 Å^3 (*ca.* 3.5% of the Cell Volume), which is likely to be occupied by a further disordered CH_3COCH_3 molecule. These voids have been treated using the SQUEEZE routine of PLATON. One Ni(CO) group displays 0.50 occupancy factor, whereas a second Ni(CO) group displays a refined occupancy factor of 0.685(3). Two $[NEt_4]^+$ cations are disordered and, thus, they have been split into two positions each and refined with one occupancy parameter per disordered group. The disordered cations have been restrained to isotropic like behaviour (ISOR line in SHELXL, s.u. 0.02) and to have similar *U* parameters (SIMU line in SHELXL, s.u. 0.02). Restraints to bond distances were applied as follow (s.u. 0.03): 1.47 Å for C–N and 1.53 Å for C–C in the disordered $[NEt_4]^+$; 1.21 Å for C–O and 1.51 Å for C–C in CH_3COCH_3.

$[NEt_4]_2[Ni_{22}P_6(CO)_{30}]\cdot 2thf$: The asymmetric unit of the unit cell contains half of a cluster anion (located on an inversion centre), one $[NEt_4]^+$ cation and one CH_3COCH_3 molecule (located on a general positions). Four Ni atoms are disordered and, thus, they have been split into two positions each and refined with one occupancy parameter per disordered group applying dummy atoms constraints (EADP line in SHELXL). Similar *U* parameter restraints have been applied to the $[NEt_4]^+$ cations and the CH_3COCH_3 molecules (SIMU line in SHELXL, s.u. 0.02).

$[NEt_4]_4[Ni_{22-x}P_2(CO)_{29-x}]\cdot 2CH_3COCH_3$($x = 0.84$): The asymmetric unit of the unit cell contains one cluster anion, four $[NEt_4]^+$ cations and two CH_3COCH_3 molecule all located on a general positions. One Ni(CO) group displays a refined occupancy factor of 0.169(9). The CH_3COCH_3 molecule have been refined isotropically. Similar *U* parameter restraints have been applied to the $[NEt_4]^+$ cations and the CH_3COCH_3 molecules (SIMU line in SHELXL, s.u. 0.01). Restraints to

bond distances were applied as follow (s.u. 0.02): 1.47 Å for C–N and 1.53 Å for C–C in $[NEt_4]^+$; 1.21 Å for C–O and 1.51 Å for C–C in CH_3COCH_3.

$[NEt_4]_6[Ni_{39}P_3(CO)_{44}]\cdot C_6H_{14}\cdot$solv: The asymmetric unit of the unit cell contains one sixth of a cluster anion located on a Wyckoff position *c* (site symmetry 3.2), half of a $[NEt_4]^+$ cation (Wyckoff position g; site symmetry .2.), one $[NEt_4]^+$ cation disordered over two equally populated (0.50 occupancy factor each) symmetry related positions (Wyckoff position g; site symmetry .2.), and one C_6H_{14} molecule disordered over six equally populated (0.16667 occupancy factor each) symmetry related positions (Wyckoff position e; site symmetry 3..). The unit cell contains an additional total potential solvent accessible void of 1159 Å^3 (*ca.* 13% of the Cell Volume), which is likely to be occupied by some further disordered C_6H_{14} molecules. These voids have been treated using the SQUEEZE routine of PLATON. Similar *U* parameter restraints have been applied to the $[NEt_4]^+$ cations and the C_6H_{14} molecules(SIMU line in SHELXL, s.u. 0.02). Restraints to bond distances were applied as follow (s.u. 0.03): 1.47 Å for C–N and 1.53 Å for C–C in $[NEt_4]^+$; 1.53 Å for C–C in C_6H_{14}.

Figure S5

ORTEP drawing of [$Ni_{14}P_2(CO)_{22}]^{2-}$ (Ni, green; P, purple; C, grey; O, red). Thermal ellipsoids are at the 30% probability level.

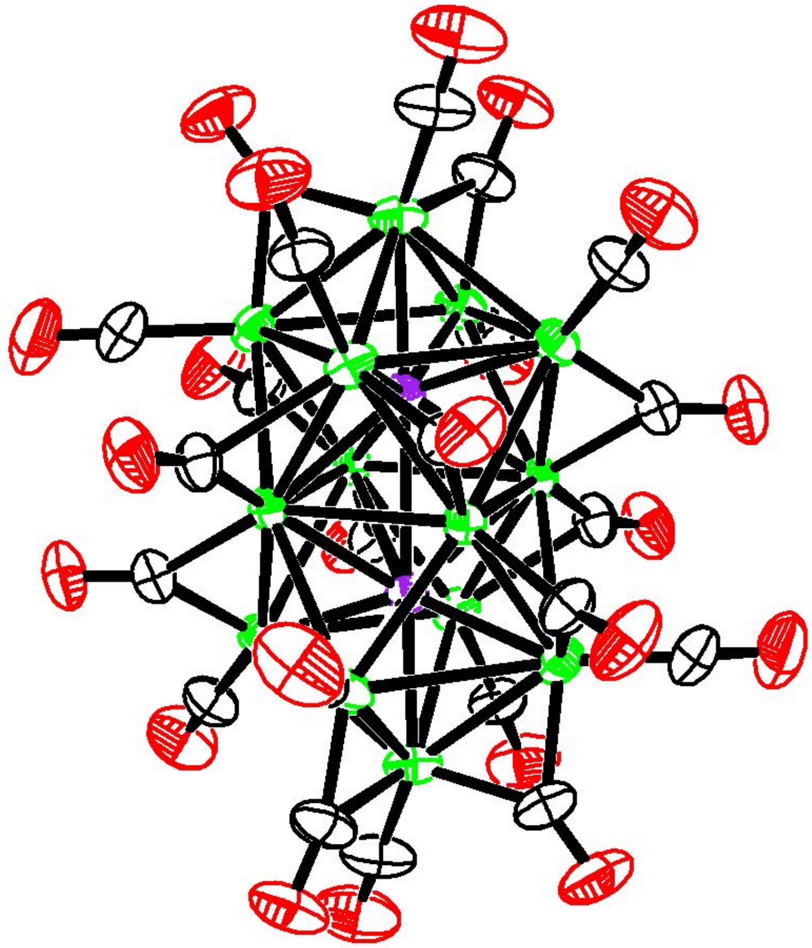

Figure S6

ORTEP drawing of $[Ni_{23-x}P_2(CO)_{30-x}]^{4-}$ *(x = 0.82) (Ni, green; P, purple; C, grey; O, red). Thermal ellipsoids are at the 30% probability level.*

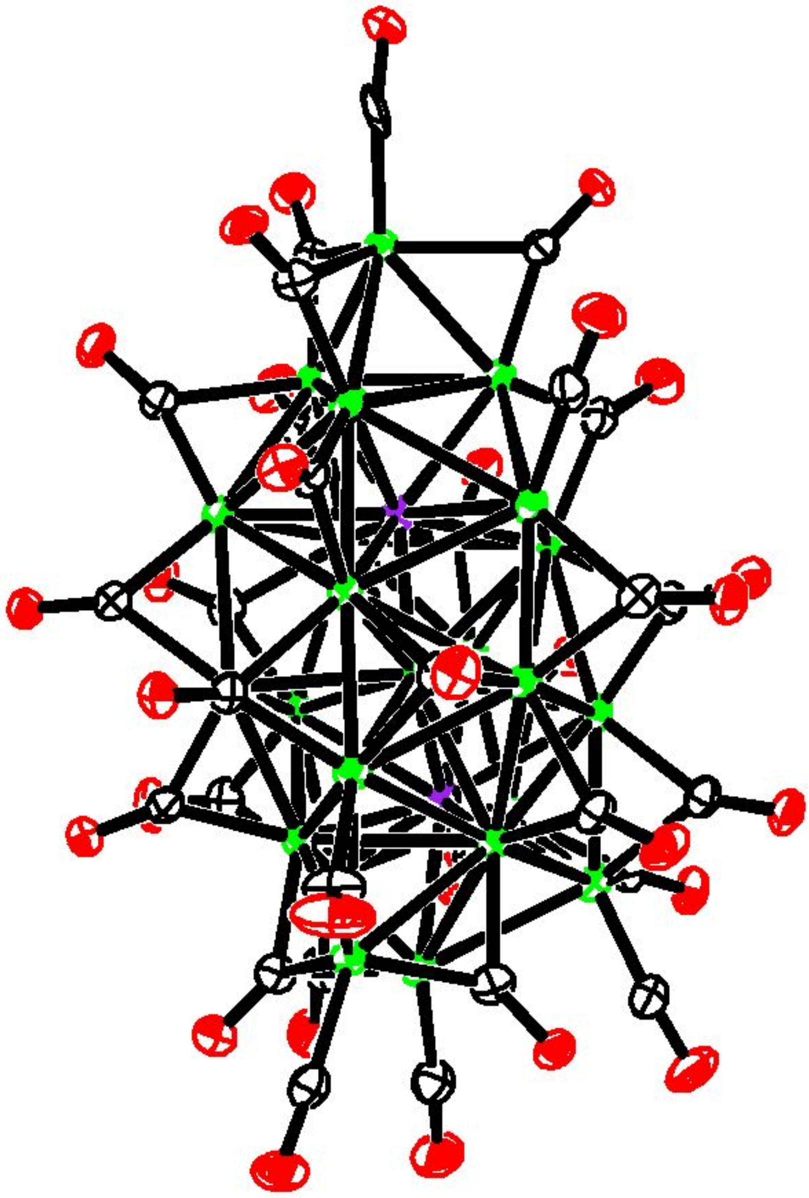

Figure S7

ORTEP drawing of [$Ni_{22}P_6(CO)_{30}]^{2-}$ (Ni, green; P, purple; C, grey; O, red). Thermal ellipsoids are at the 30% probability level.

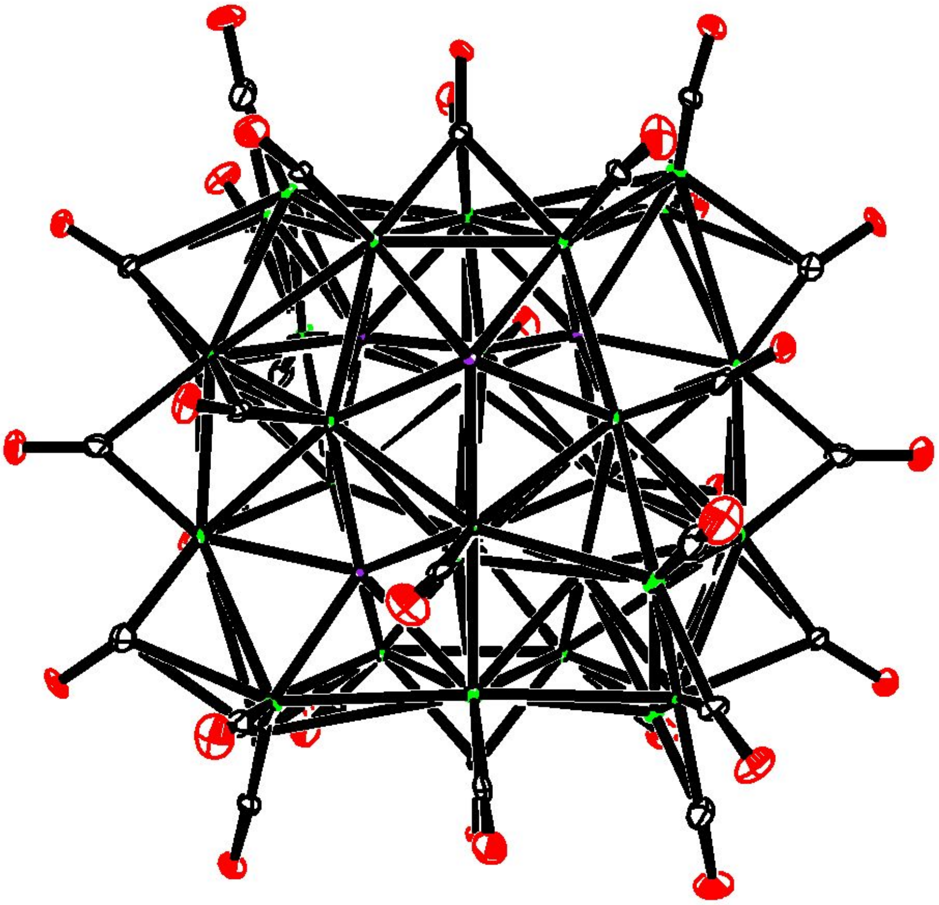

Figure S8

ORTEP drawing of $[Ni_{22-x}P_2(CO)_{29-x}]^{4-}$ *(x = 0.84) (Ni, green; P, purple; C, grey; O, red). Thermal ellipsoids are at the 30% probability level.*

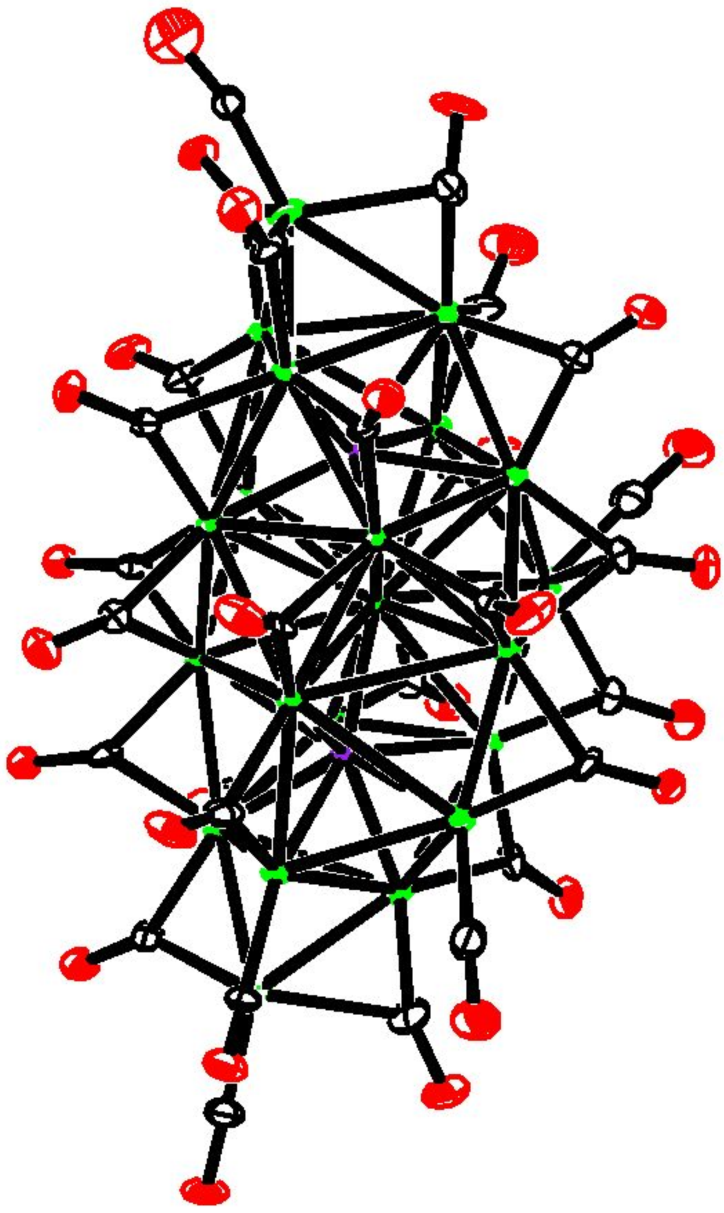

Figure S9

ORTEP drawing of $[Ni_{39}P_3(CO)_{44}]^{6-}$ (Ni, green; P, purple; C, grey; O, red). Thermal ellipsoids are at the 30% probability level.

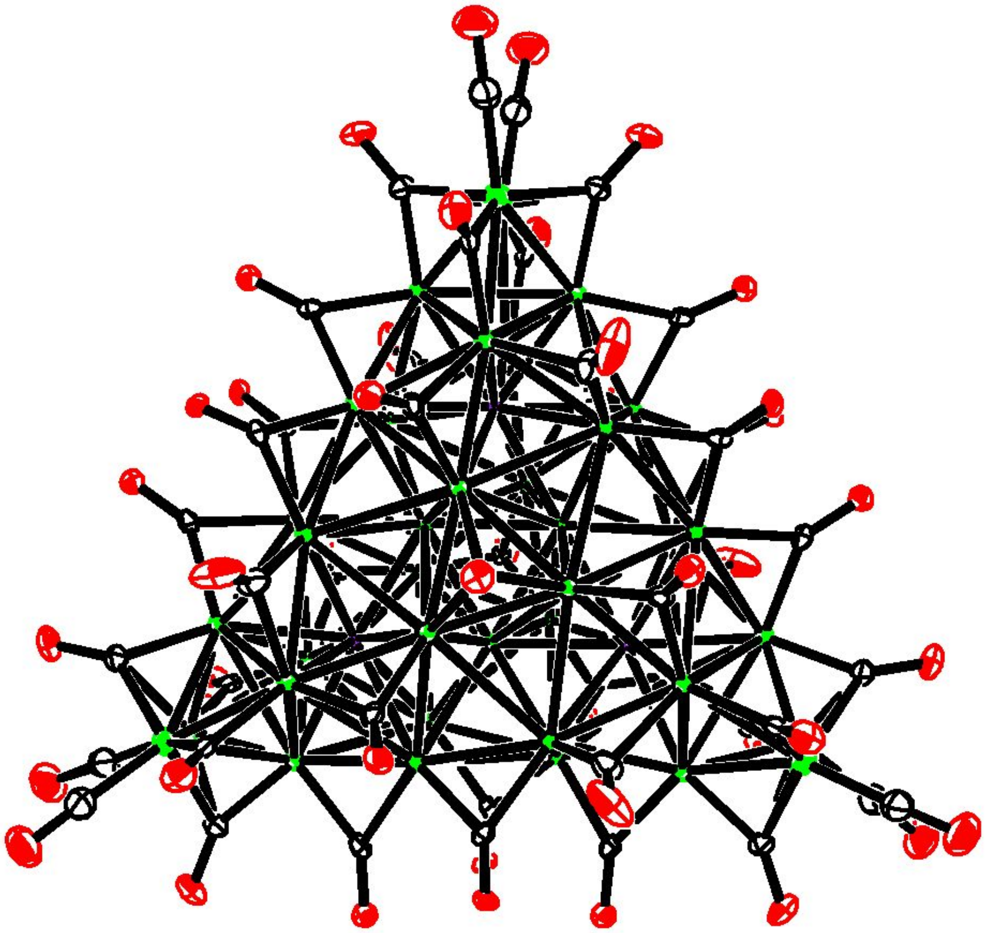

Supplement: Supplementary file 1 — ic0c02572_si_001.pdf [file ic0c02572_si_001.pdf]
